# Synthesis of an Indazole/Indazolium Phosphine Ligand Scaffold and Its Application in Gold(I) Catalysis

**DOI:** 10.1021/acs.organomet.3c00354

**Published:** 2023-09-18

**Authors:** Asima Munawar, Logan T. Maltz, Wei-Chun Liu, François P. Gabbaï

**Affiliations:** Department of Chemistry, Texas A&M University, College Station, Texas 77843, United States

## Abstract

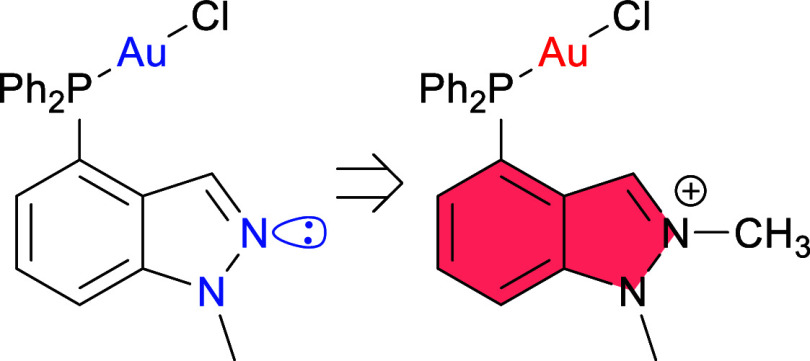

Advances in ligand development have allowed for the fine-tuning of gold catalysis. To contribute to this field, we designed an indazole phosphine ligand scaffold that allows facile introduction of cationic charge through methylation. With minimal changes to the structure upon methylation, we could assess the importance of the electronic effects of the insertion of a positive charge on the catalytic activity of the resulting gold(I) complex. Using the benchmark reactions of propargyl amide cyclization and enyne cyclization with and without hexafluoroisopropanol (HFIP), we observed marked differences in the catalytic activities of the neutral and cationic gold species.

Phosphines are common ancillary ligands in gold catalysis. These ligands’ properties are easily tuned by chemical modification, allowing for simple yet impactful changes to catalytic activity.^1^ One method for modification that has seen increasing use is the insertion of a positive charge in the ligand backbone.^2^ This positive charge has differing impacts on the coordinated metal depending on its position. Over the past few years, the Gabbaï group has synthesized a group of acridinium- (**A**) and xanthylium-based (**B**) γ-cationic phosphines that position the cationic charge at a remote position to withdraw electron density orthogonally from gold while minimizing the reduction in σ-donation from the phosphine (Figure 1).^3^ Prior to these developments, several groups investigated α-cationic phosphines—including phosphines of types **C**(4) and **D**(5)—wherein electron density is withdrawn directly from P, thereby weakening σ-donation to the attached gold (Figure 1).

**Figure 1:**
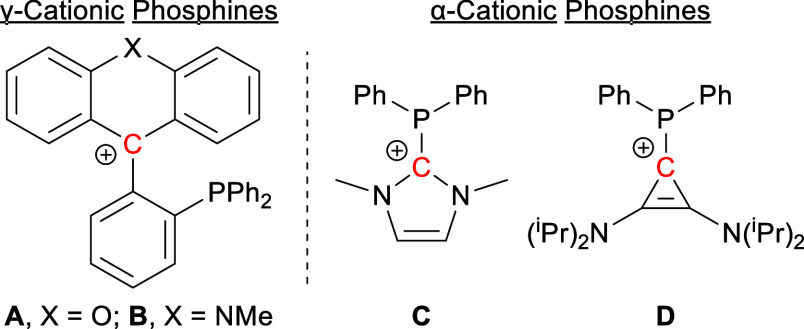
Examples of γ- and α-cationic phosphines.

This method produces a phosphine that is as poor of a donor as PF_3_, P(CF_3_)_3_, and PCl_3_ while avoiding the air- and moisture-sensitivity typical of the phosphorus-halogen bond.^2c^ Due to their electron-poor nature, these cationic phosphines have been commonly used to promote reactions catalyzed by electron-rich transition metals where the catalytic step benefits from a more electrophilic metal center, a common example being the gold(I) activation of alkenes, allenes, and alkynes.^6^ Because of the minimal back-bonding from gold, a balance must be found between maximizing the electrophilicity of gold through reduction of σ-donation from the phosphine while avoiding decomposition of the catalyst.

Adding to the collection of cationic phosphines, we report the synthesis of a new phosphine ligand containing an indazole group directly bound to the phosphorus, which allows for facile insertion of a positive charge through methylation of the indazole backbone. A similar approach was taken in 2008 by Debono et al. for modifying a bisphosphine for application in palladium catalysis.^7^ This simple method for positive-charge insertion allows us to easily modulate the donicity and directly compare the newly synthesized neutral and cationic phosphine gold(I) complexes. We look to the common benchmark reactions of propargyl amide cyclization and enyne cyclization to assess the differences in their reactivity.

Building on our recent efforts in the chemistry of indazole-based ligands,^8^ we decided to investigate the introduction of a phosphine functionality at the 4-position of this compound. To this end, ^n^BuLi was added to a solution of 4-bromo-1-methyl-1*H*-indazole (**1**) in dry tetrahydrofuran (THF) at −78 °C. After stirring for 30 min, Ph_2_PCl was added as a phosphorus source. The resulting mixture was warmed to room temperature and stirred overnight to give **2** as a pale-yellow solid (Figure 2). The formation of **2** is easily followed by ^31^P NMR spectroscopy, which shows a single peak at −11.84 ppm. ^1^H NMR spectroscopy further evinces the phosphine’s formation with a diagnostic singlet for the indazole C-*H* at 7.62 ppm in addition to a distinct methyl peak at 4.02 ppm. Layering of hexanes over a dichloromethane (DCM) solution of **2** yielded clear block crystals, and single-crystal X-ray diffraction (SCXRD) further established the identity of **2** (Figure S1).

**Figure 2:**
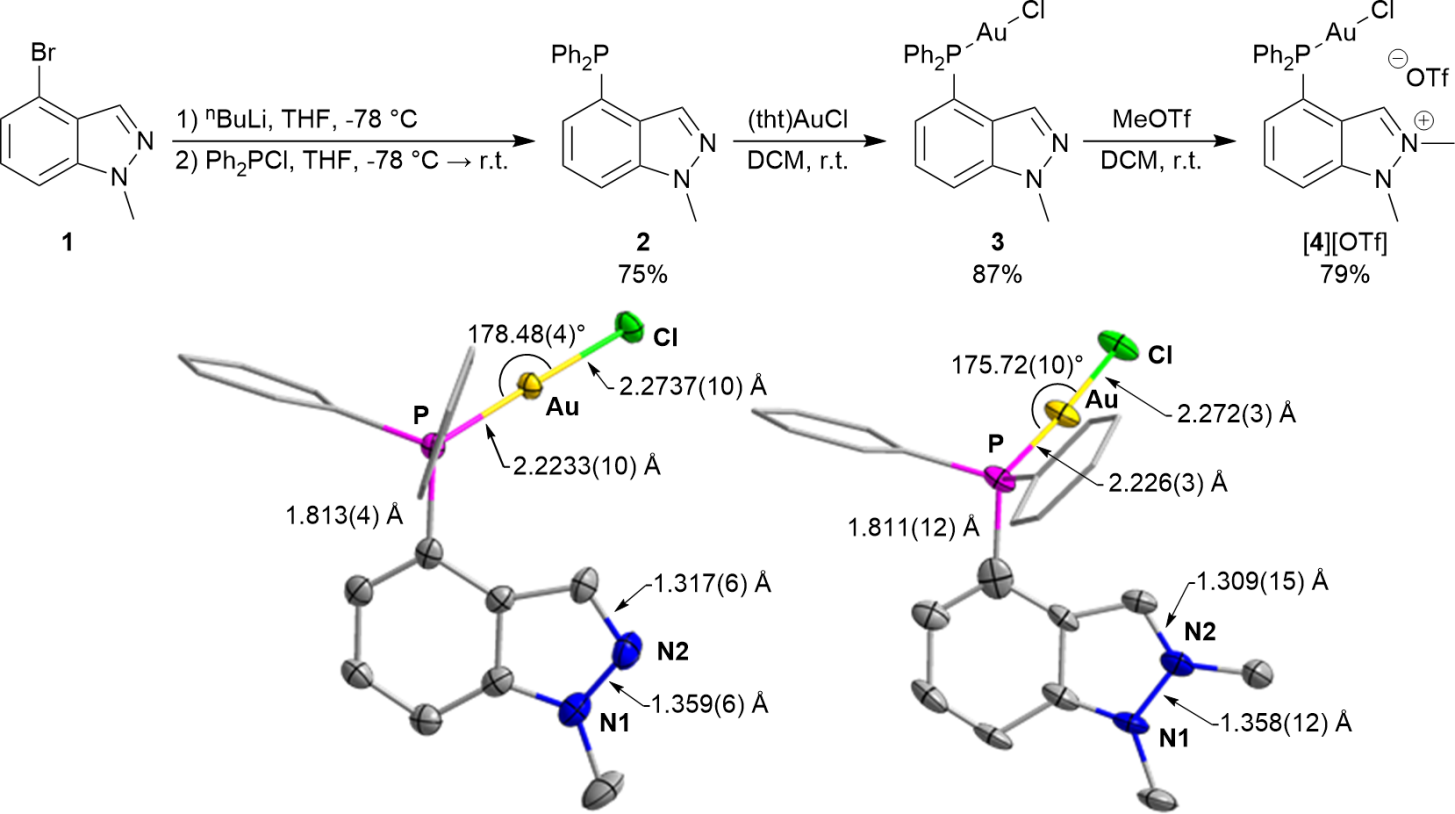
(top) Reaction scheme for synthetic procedures. (bottom) Crystal structures of **3** (left) and [**4**][OTf] (right) in the solid state showing selected bond lengths and angles. Hydrogen atoms, solvent, and anions omitted for clarity. Thermal ellipsoids drawn at 50% probability, and phenyl groups drawn as thin lines.

We combined **2** with 1.1 equiv of (tht)AuCl (tht = tetrahydrothiophene) in DCM at room temperature. After stirring for 30 min, the resulting solution was concentrated to 1 mL before hexanes were added to precipitate **3** as a white powder. A peak at 26.06 ppm in the ^31^P NMR—downfield of the peak corresponding to the free ligand—confirmed the formation of **3**. Slow diffusion of hexanes into a concentrated solution of **3** in DCM gave clear plate crystals which were analyzed by SCXRD (Figure 2).

With the phosphine now protected by gold, we were able to methylate the indazole nitrogen by reacting **3** with 1.1 equiv of methyl trifluoromethanesulfonate (MeOTf) in dry DCM under inert atmosphere. The mixture was stirred overnight, and light yellow [**4**][OTf] was precipitated using hexanes. The formation of this cationic species was readily verified by NMR spectroscopy, which showed an upfield shift in the ^31^P NMR spectrum from 26.06 to 24.97 ppm. In the ^1^H NMR spectrum, the indazolium proton singlet saw a significant downfield shift from 7.83 to 8.70 ppm, accompanied by an increase in the integral of the methyl peak by three protons due to merging of the new methyl peak with the original one. X-ray quality clear block crystals were obtained by slow diffusion of Et_2_O into a concentrated solution of [**4**][OTf] in acetonitrile (Figure 2).

Comparing the crystal structures of **3** and [**4**][OTf] in Figure 2, there is not a significant difference in either the bond lengths or the bond angles. This minimal perturbation of the structure upon introduction of positive charge has been previously observed in the case of pyridine/pyridinium phosphine gold(I) complexes.^9^ Despite this lack of structural change, we expected to see more significant differences in their catalytic activities; therefore, we turned to the benchmark reactions of propargyl amide cyclization and enyne cyclization.

We started our catalytic investigations with the cyclization of *N*-propargyl-4-fluorobenzamide, a reaction commonly used to gauge the activity of gold catalysts.^3b,10^ Silver salts are often used to activate the otherwise stable Au–Cl bond to access the cationic gold species that serves as the active catalyst. This activation promotes substrate binding not only by opening a coordination site on gold but also by increasing the metal center’s Lewis acidity.^11^ Aside from the drawbacks of adding silver salts to the reaction mixture,^11^ the already increased Lewis acidity of the gold center in our cationic system due to the weakly donating phosphine led us to consider a milder activator—hexafluoroisopropanol (HFIP).

HFIP as a solvent has been increasingly employed to facilitate a range of catalyses^12^ and has even found its way into the field of gold catalysis.^13^ Many of these gold catalysts use HFIP simply as a polar protic solvent with the catalyst still being activated by a silver salt. Some of the more recent examples, however, use HFIP not only to solubilize the catalyst but also to activate the Au–Cl bond by hydrogen bonding with chloride.^13c,13d,13f−13h^ While these groups favored HFIP as the primary solvent for their reactions, due to solubility, we decided to use HFIP more as an additive with CDCl_3_ being the solvent.

Using a 1:11 HFIP/CDCl_3_ solution, 2 mol% catalyst loading yielded decent conversion of **5** within a reasonable amount of time. The progress of the reaction was monitored by ^1^H NMR spectroscopy, and the results are summarized in Table 1.

**Table 1:**
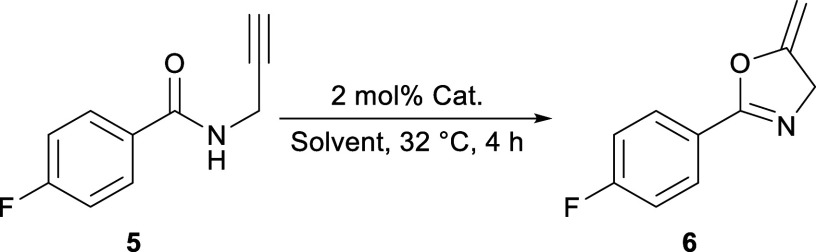
Propargyl Amide Cyclization

Entry	Cat.	Solvent	Conversion (%)^a^
1	Ph_3_PAuCl	1:11 HFIP/CDCl_3_^b^	13
2	**3**	1:11 HFIP/CDCl_3_^b^	22
3	[**4**][OTf]	1:11 HFIP/CDCl_3_^b^	89
4	Ph_3_PAuCl	CDCl_3_	0
5	**3**	CDCl_3_	100^c^
6	[**4**][OTf]	CDCl_3_	d

a Conversion determined by ^1^H NMR.

b vol/vol ratio.

c Reaction complete after 2 h.

d [**4**][OTf] insoluble in CDCl_3_.

Under these conditions, the reaction saw only a 13% conversion within 4 h using Ph_3_PAuCl (entry 1), a significant drop compared to Tzouras et al.’s complete conversion of *N*-propargyl benzamide within 3 h using pure HFIP and a 1 mol% catalyst loading.^13g^ This result suggests that increasing the ratio of HFIP to CDCl_3_ increases the catalytic activity. Even so, cationic [**4**][OTf] achieved 89% conversion under these same conditions (entry 3). The reaction only proceeded to 22% conversion in the presence of **3**, which was expected given the similar structures of **3** and Ph_3_PAuCl around the P (entry 2). Taken together, this data illustrates that by decreasing the σ-donation from the phosphine, the Lewis acidity of gold—and thus the catalytic activity—increases. As is typical for any catalytic study, we needed to verify that the additive, HFIP, does indeed promote this reaction.

Therefore, we performed the reaction without the addition of HFIP under the same experimental conditions. Because Au–Cl species are typically seen as precatalysts requiring activation, we expected to see no reactivity without HFIP. To our surprise, while the reaction no longer proceeded with Ph_3_PAuCl (entry 4), complete conversion was observed within 2 h using neutral **3** as the catalyst (entry 5). This substantial increase in catalytic activity without HFIP indicates a different process at work than we initially assumed. A notable difference between PPh_3_ and **2** as ligands is the presence of a free lone pair on the nitrogen of the indazole of **2**. This nitrogen may act as a Brønsted base during the reaction, perhaps promoting the deprotonation of the propargyl amide starting material—producing a stronger nucleophile—or assisting in the protodeauration step. In the presence of HFIP, this hydrogen bond accepting site is likely quenched, resulting in decreased catalytic activity as **2** becomes like PPh_3_ again, only contacting the catalytic cycle through the P atom. Unfortunately, we were unable to directly compare the activities of **3** and [**4**][OTf] without HFIP due to the insolubility of the cation in pure CDCl_3_ (entry 6), highlighting HFIP’s role as not only an activator but also a solubilizing agent for polar catalysts. We did attempt the catalysis in CD_3_CN—which did dissolve [**4**][OTf]—but the reactivity was minimal, even in the presence of HFIP.

With this understanding of the reactivity of **3**, we wanted to test a reaction in which there were no acidic protons, allowing us to focus our comparison on the change in the donation of electron density to the gold upon introducing a positive charge. As such, we turned to the cyclization of 2-allyl-2-(2-propynyl)malonate, which lacks an acidic proton. As this reaction is known to be more challenging, we increased the HFIP concentration to 1:2 HFIP/CDCl_3_ and the catalyst loading to 5 mol% (Table 2). Even under these conditions, neither Ph_3_PAuCl nor neutral **3** promoted cyclization (entries 1 and 2). Catalyst [**4**][OTf], on the other hand, facilitated 86% conversion within four hours as indicated by ^1^H NMR spectroscopy (entry 3). Without HFIP, the reaction did not proceed with any of the catalysts, indicating the necessity of this additive for the success of this reaction (entries 4–6). The higher activity of [**4**][OTf] can be attributed to the weaker σ-donation of the phosphine enhancing the electrophilic character of the gold center.

**Table 2:**
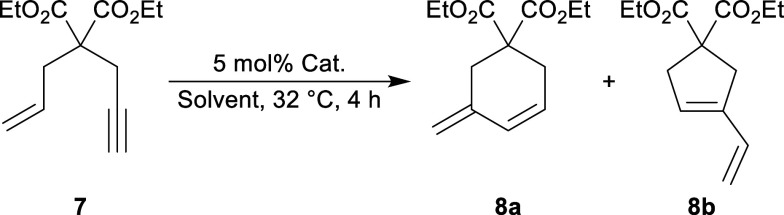
Enyne Cyclization

Entry	Cat.	Solvent	Conversion (%)^a^
1	Ph_3_PAuCl	1:2 HFIP/CDCl_3_^b^	0
2	**3**	1:2 HFIP/CDCl_3_^b^	0
3	[**4**][OTf]	1:2 HFIP/CDCl_3_^b^	86^c^
4	Ph_3_PAuCl	CDCl_3_	0
5	**3**	CDCl_3_	0
6	[**4**][OTf]	CDCl_3_	d

a Conversion determined by ^1^H NMR.

b vol/vol ratio.

c **8a**/**8b** = 6:1.

d [**4**][OTf] insoluble in CDCl_3_.

In summary, we synthesized a new phosphine ligand containing an indazole group. After complexation with gold, the lone pair on nitrogen allowed for easy introduction of a positive charge through methylation, thereby permitting the straightforward comparison of neutral and cationic gold phosphine complexes with minimal structural changes. We used the common reporter reactions of propargyl amide cyclization and enyne cyclization to assess the differences between these two new complexes. In both reactions, the cationic species outperforms the neutral species in solutions containing HFIP as an additive, demonstrating the benefits of decreasing the level of σ-donation as a way to increase the Lewis acidity of gold. In pure CDCl_3_, however, the neutral complex performed even better in the propargyl amide cyclization than the cationic one in the HFIP/CDCl_3_ solution, suggesting that in this particular reaction, the Brønsted basic nitrogen of the neutral catalyst also participates. This result reminds us that HFIP is still an additive and, like silver, can engage the reactive species productively or counterproductively depending on the specifics of the reaction mixture.

## Supporting Information Available

Synthetic methods, NMR spectra, and details and NMR spectra for catalytic studies (PDF)
