# Supplementary material for: Synthesis of an Indazole/Indazolium Phosphine Ligand Scaffold and Its Application in Gold(I) Catalysis
Source: Organometallics. 2023 Sep 18;42(19):2742–6. doi: 10.1021/acs.organomet.3c00354 (PMC10863396; doi:10.1021/acs.organomet.3c00354)
Supplement: Supplementary file 1 — om3c00354_si_001.pdf [file om3c00354_si_001.pdf]

Supporting Information for

Synthesis of an Indazole/Indazolium Phosphine Ligand Scaffold and its Application in Gold(I) Catalysis

Asima Munawar,[a] Logan T. Maltz,[a] Wei-Chun Liu,[a] and François P. Gabbaï*,[a]

[a]*Department of Chemistry, Texas A&M University, College Station, Texas 77843, United States*

**Email: francois@tamu.edu*

Table of Contents

Experimental 3
Synthesis of 2 4
Crystal Structure of 2 5
NMR Spectra of 2 6
Synthesis of 3 9
NMR Spectra of 3 10
Synthesis of [4][OTf] 13
NMR Spectra of [4][OTf] 14
Catalytic Studies 18
Procedure for Propargyl Amide Cyclization Studies 18
Stacked Spectra for Propargyl Amide Cyclization Studies 19
Procedure for Enyne Cyclization Studies 24
Stacked Spectra for Enyne Cyclization Studies 25
References 30

Experimental

General considerations

4-Bromo-1-methyl-*1H*-indazole[1] **1** and (tht)AuCl (tht = tetrahydrothiophene)[2] were prepared by following literature methods. All other chemicals were purchased from commercial chemical suppliers and used as received. Solvents were dried by refluxing under N_2 over Na/K (THF) or CaH_2 (DCM and acetonitrile) and being stored over molecular sieves in the glovebox. All other solvents were ACS reagent grade and used as received. ^{1}H and ^{31}P NMR spectra were recorded on a Bruker Avance 400 spectrometer at 298.0 K (400.20 MHz for ^{1}H; 162.00 MHz for $^{31}P\{^{1}H\}$) and ^{1}H and ^{13}C spectra were recorded on a Bruker Avance 500 NMR spectrometer at 305.0 K (500.13 MHz for ^{1}H; 125.77 MHz for $^{13}C\{^{1}H\}$) equipped with an automated tuning 5 mm $^{1}H/^{13}C/^{15}N$ cold probe. Chemical shifts are given in ppm and are referenced to residual solvent signals ^{1}H ($CHCl_3$: 7.26 ppm; CHD_2CN: 1.94 ppm) or ^{13}C ($CDCl_3$: 77.16 ppm; CD_3CN: 1.32 ppm) or external 85% H_3PO_4 (^{31}P). Elemental analyses were performed at Atlantic Microlab (Norcross, GA).

Crystallographic measurements

Crystallographic measurements were made on Bruker D8 QUEST diffractometer at 110 K using a Bruker D8 QUEST diffractometer (Mo-Kα radiation, = 0.71073 Å) equipped with a Photon III detector. A suitable single crystal was selected and mounted onto a nylon loop in each case. Integrated intensity information for each reflection was obtained by reducing the data frames with the program APEX4.[3] The semi-empirical method SADABS was used for absorption corrections.[4] The structures were solved by intrinsic phasing (ShelXT)[5] and refined by the full-matrix least-square technique against F^2 with anisotropic temperature-dependent parameters for all non-hydrogen atoms (ShelXL)[13] using the Olex2-1.5 interface.[6] All hydrogen atoms were geometrically placed and refined using the riding atom model. Diamond4 was employed for the final data presentation and structure plots. The data has been deposited with the Cambridge Structural Database. CCDC 2288242-2288244 contain the supplementary crystallographic data for this paper.

Synthesis of 2

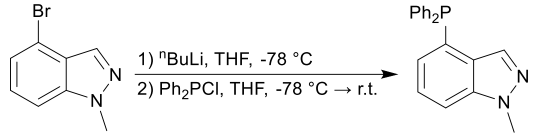

1 2

n-Butyllithium (3.22 mL, 8.1 mmol, 2.5 M in hexanes) was added to a solution of 4-bromo-1-methyl-*1H*-indazole **1** (1.7010 g, 8.0593 mmol) in THF (50 mL) at -78 ˚C, leading to a color change from pale yellow to bright orange. The resulting solution was stirred at that temperature for 30 minutes at which point chlorodiphenylphosphine (1.7778 g, 8.0576 mmol) was added dropwise with continuous stirring. The reaction mixture was warmed to room temperature and stirred overnight, then quenched by adding a saturated aqueous ammonium chloride solution (40 mL). After removing the THF via rotary evaporation, the remaining aqueous phase was extracted with CH_2Cl_2 (3 × 30 mL). The organic fractions were combined, dried over $MgSO_4$, and removed *in vacuo* to give a yellow oil. This oil was triturated with hexanes, producing a pale-yellow powder which was collected by filtration and dried to afford **2** (1.9060 g, 6.0252 mmol, 75% yield). Single crystals of **2** were obtained as colorless plates by the slow diffusion of hexanes into a CH_2Cl_2 solution of the compound. **^{1}H NMR** (500.13 MHz, CD_3CN, 305.0 K) δ (ppm) 7.62 (s, 1H, indazole-N=C*H*), 7.53 (d, *J* = 8.5 Hz, 1H, indazole-*H*), 7.41-7.32 (m, 11H, 10 from Ph-*H* and 1 from indazole-*H*), 6.82 (t, *J* = 13.9 Hz, 1H, indazole-*H*), 4.02 (s, 3H, N-C*H*$_3$). **^{13}C{^{1}H} NMR** (125.77 MHz, CD_3CN, 305.0 K) δ (ppm) 140.44 (d, *J* = 6.9 Hz, *N*-*ipso*-indazole-*C*), 137.09 (d, *J* = 10.4 Hz, indazole-*C*), 134.77 (d, *J* = 20.4 Hz, *o*-Ph-*C*), 132.74 (d, *J* = 8.0 Hz, indazole-N=*C*H), 131.70 (d, *J* = 14.1 Hz, *ipso*-indazole-*C*), 130.19 (s, *p*-Ph-*C*), 129.72 (d, *J* = 7.2 Hz, *m*-Ph-*C*), 127.20 (d, *J* = 21.8 Hz, *ipso*-Ph-*C*), 126.95 (d, *J* = 4.0 Hz, indazole-*C*), 126.45 (d, *J* = 9.1 Hz, indazole-*C*), 111.28 (s, indazole-*C*), 36.21 (s, N-*C*H$_3$). **^{31}P{^{1}H} NMR** (161.95 MHz, CD_3CN, 298.0 K) δ (ppm) **-**11.84 (s). **Elemental Analysis:** $C_{20}H_{17}N_2P$: Calculated: C, 75.94; H, 5.42; N, 8.86; Found: C, 76.07; H, 5.31; N, 8.89.

Crystal Structure of 2

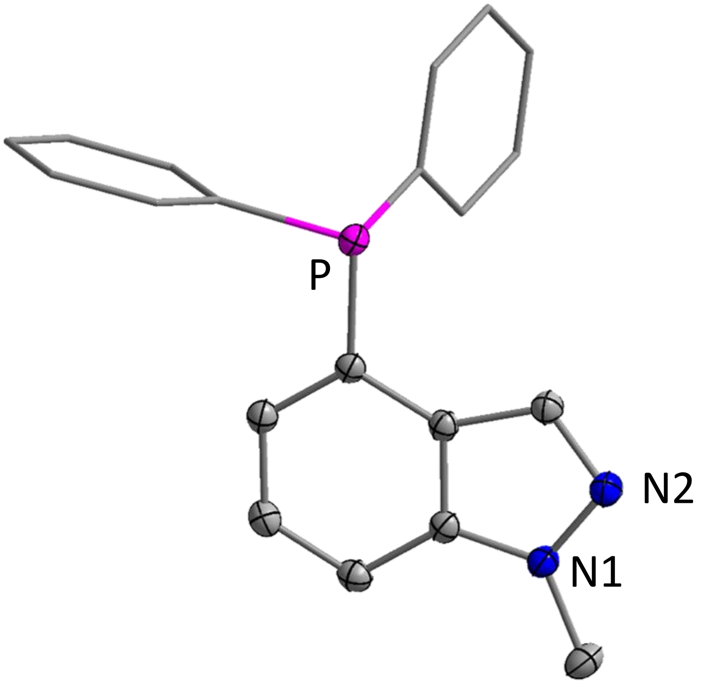

Figure S1. Crystal structure of **2**. Hydrogen atoms omitted for clarity. Thermal ellipsoids drawn at 50% probability, and phenyl groups drawn as thin lines (gray: C, blue: N, magenta: P).

NMR Spectra of 2

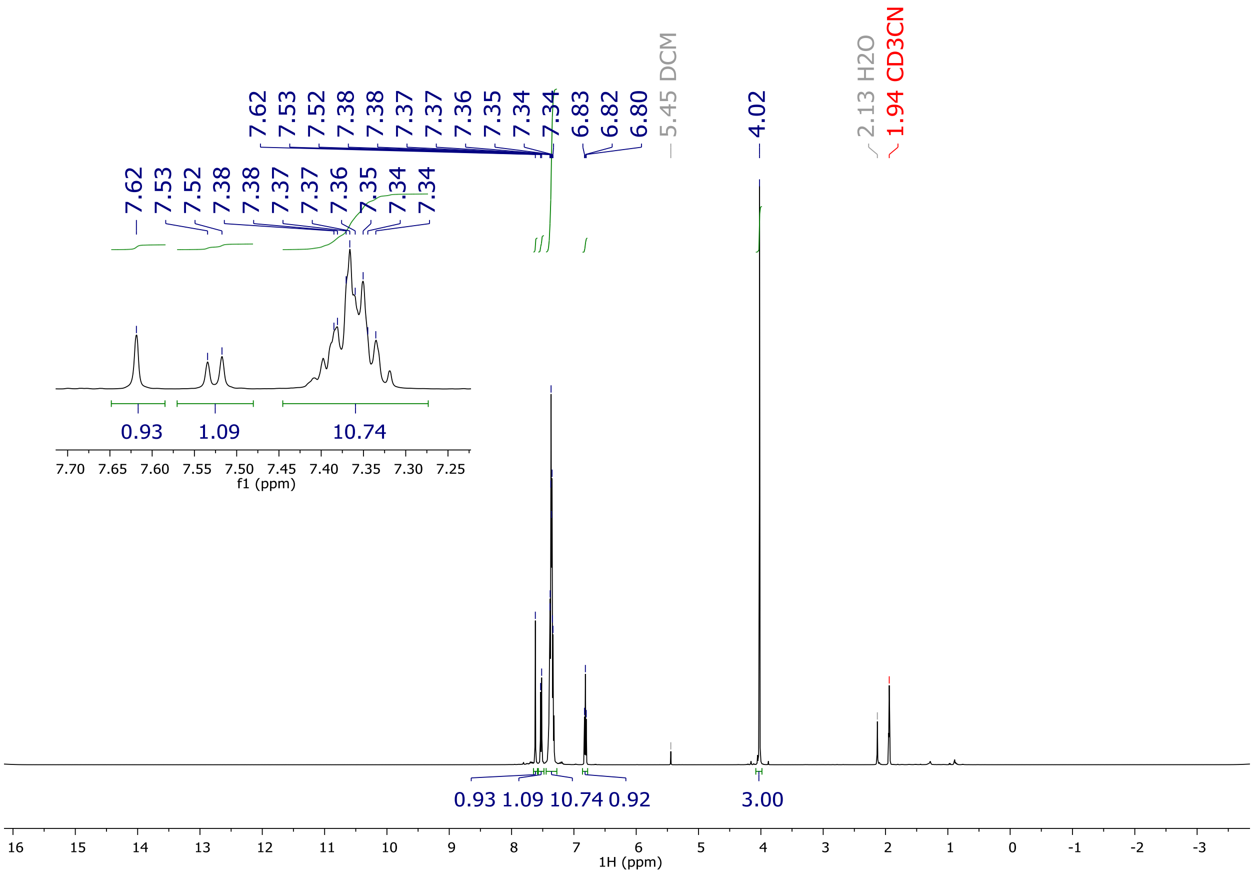

Figure S2. ^{1}**H NMR** (500.13 MHz, CD_3CN) spectrum of **2**.

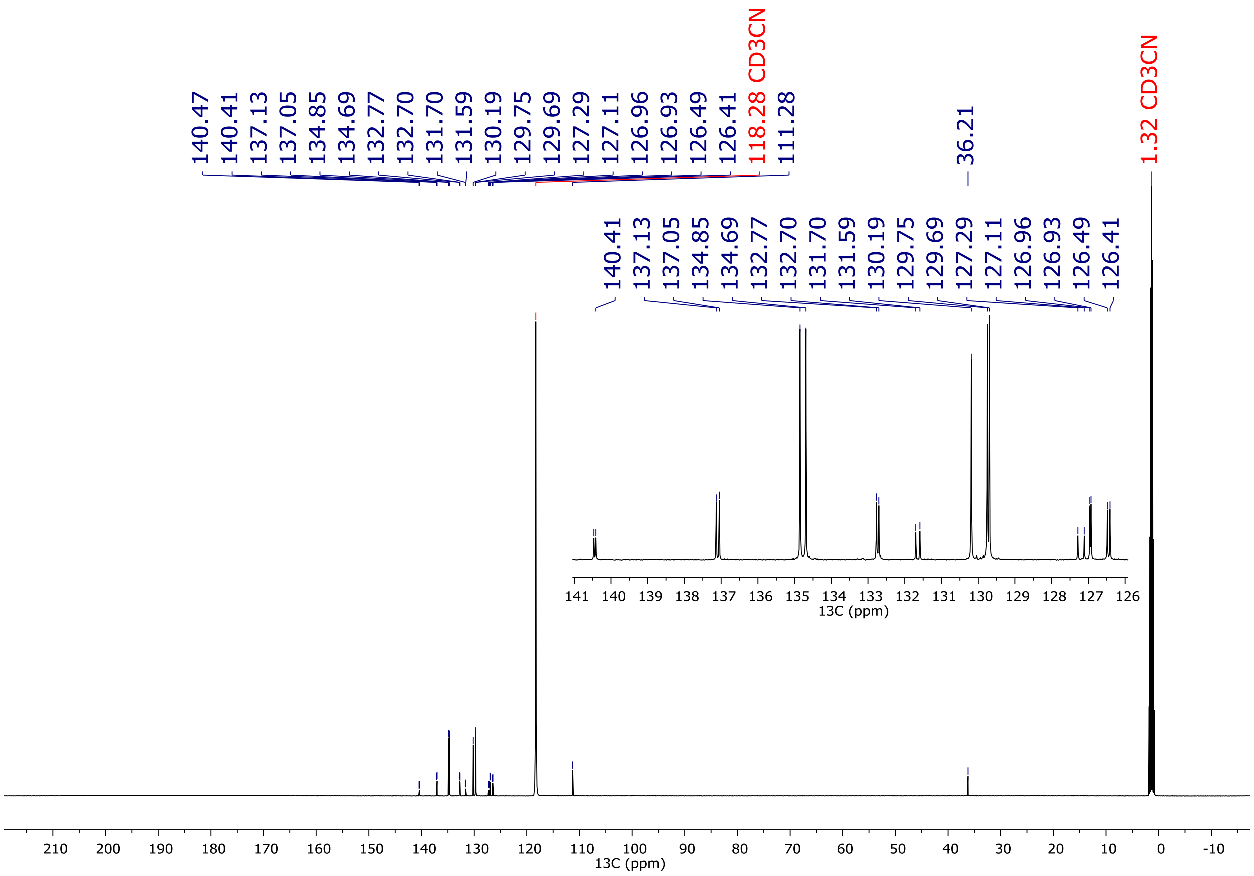

Figure S3. **$^{13}C\{^1H\}$ NMR** (125.77 MHz, CD_3CN) spectrum of **2**.

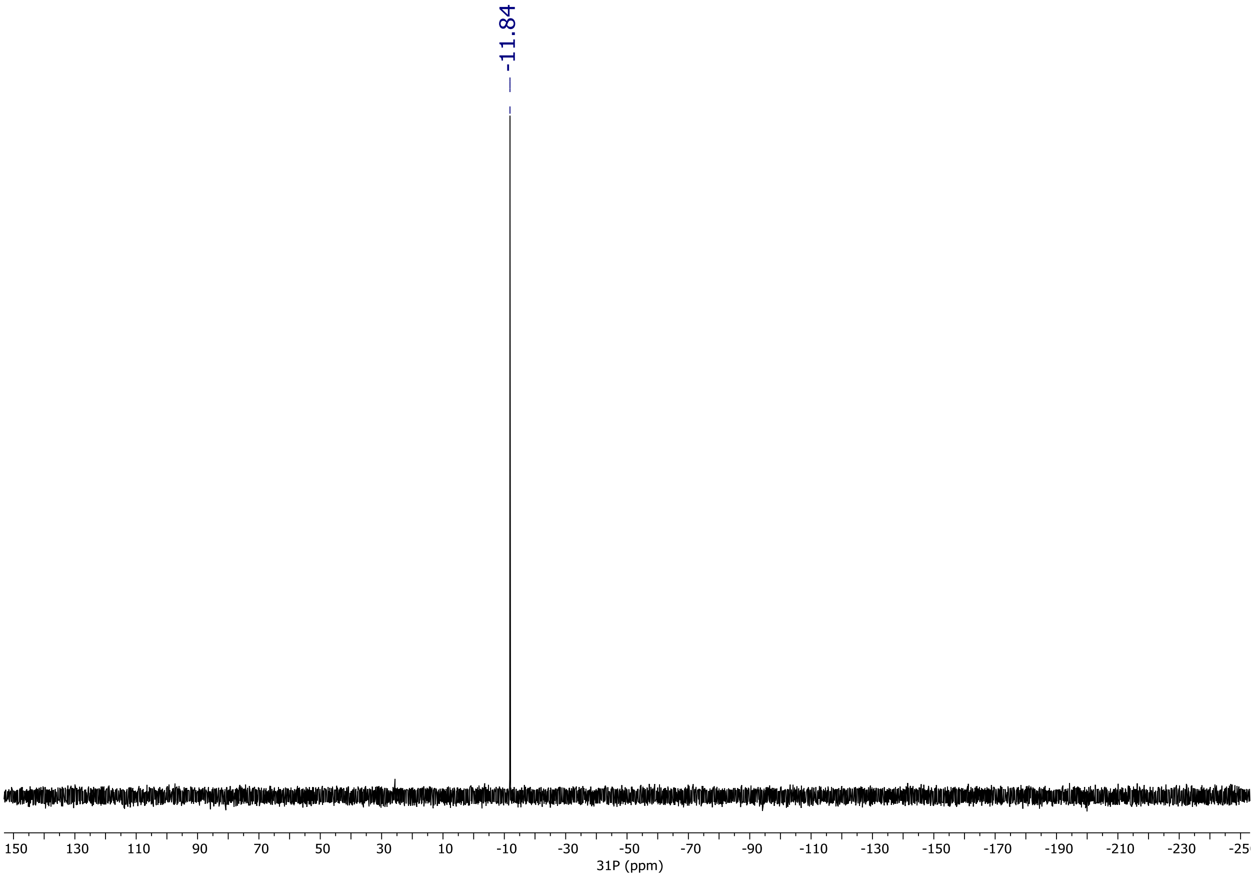

Figure S4. ^{31}**P{**^{1}**H} NMR** (161.95 MHz, CD_3CN) spectrum of **2**.

Synthesis of 3

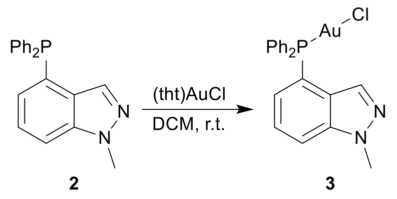

(tht)AuCl (0.6256 g, 1.951 mmol) was added to a solution of **2** (0.6010 g, 1.900 mmol) in DCM (15 mL) at room temperature. The resulting solution was stirred for 30 minutes and then triturated with hexanes followed by filtration and drying to afford **3** as a white powder (0.9075 g, 1.654 mmol, 87% yield). Single crystals of **3** were obtained as colorless plates by the slow diffusion of hexanes into a CH_2Cl_2 solution of the compound. **^{1}H NMR** (400.20 MHz, CD_3CN, 298.0 K) δ (ppm) 7.83 (s, 1H, indazole-N=C*H*), 7.77 (dd, *J* = 8.6, 2.4 Hz, 1H, indazole-*H*), 7.67 – 7.62 (m, 6 H, Ph-*H*), 7.57 – 7.53 (m, 4H, Ph-*H*), 7.46 (m, 1H, indazole-*H*), 6.98 (dd, *J* = 14.5, 7.0 Hz, 1H, indazole-*H*), 4.08 (s, 3H, N-C*H*$_3$). **^{13}C{^{1}H} NMR** (125.77 MHz, CD_3CN, 305.0 K) δ (ppm) 140.81 (d, *J* = 10.9 Hz, *N*-*ipso*-indazole-*C*), 135.39 (d, *J* = 14.1 Hz, *o*-Ph-*C*), 133.49 (d, *J* = 2.7 Hz, *p*-Ph-*C*), 131.94 (d, *J* = 6.0 Hz, indazole-N=*C*H), 130.54 (d, *J* = 12.0 Hz, *m*-Ph-*C*), 128.74 (d, *J* = 63.8 Hz, *ipso*-Ph-*C*), 128.36 (d, *J* = 9.1 Hz, indazole-*C*), 126.82 (d, *J* = 11.7 Hz, indazole-*C*), 125.23 (d, *J* = 13.7 Hz, indazole-*C*), 122.17 (d, *J* = 62.3 Hz, *ipso*-indazole-*C*), 115.02 (d, *J* = 2.7 Hz, indazole-*C*), 36.48 (s, N-*C*H$_3$). **^{31}P{^{1}H} NMR** (162.00 MHz, CD_3CN, 298.0 K) δ (ppm) 26.06 (s). **Elemental Analysis:** $C_{20}H_{17}AuClN_2P$: Calculated: C, 43.78; H, 3.12; N, 5.10; Found: C, 43.67; H, 3.31; N, 5.01.

NMR Spectra of 3

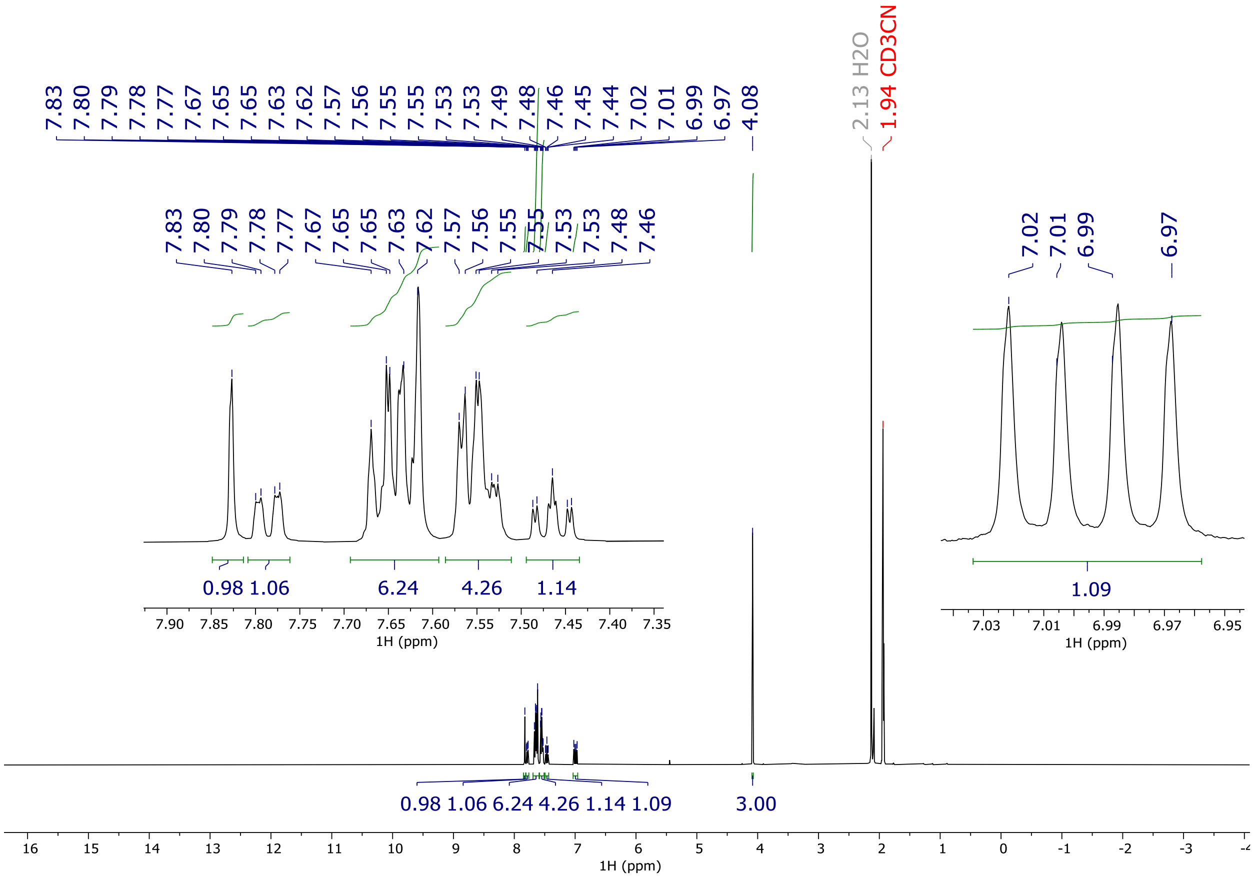

Figure S5. ^{1}**H NMR** (400.20 MHz, CD_3CN) spectrum of **3**.

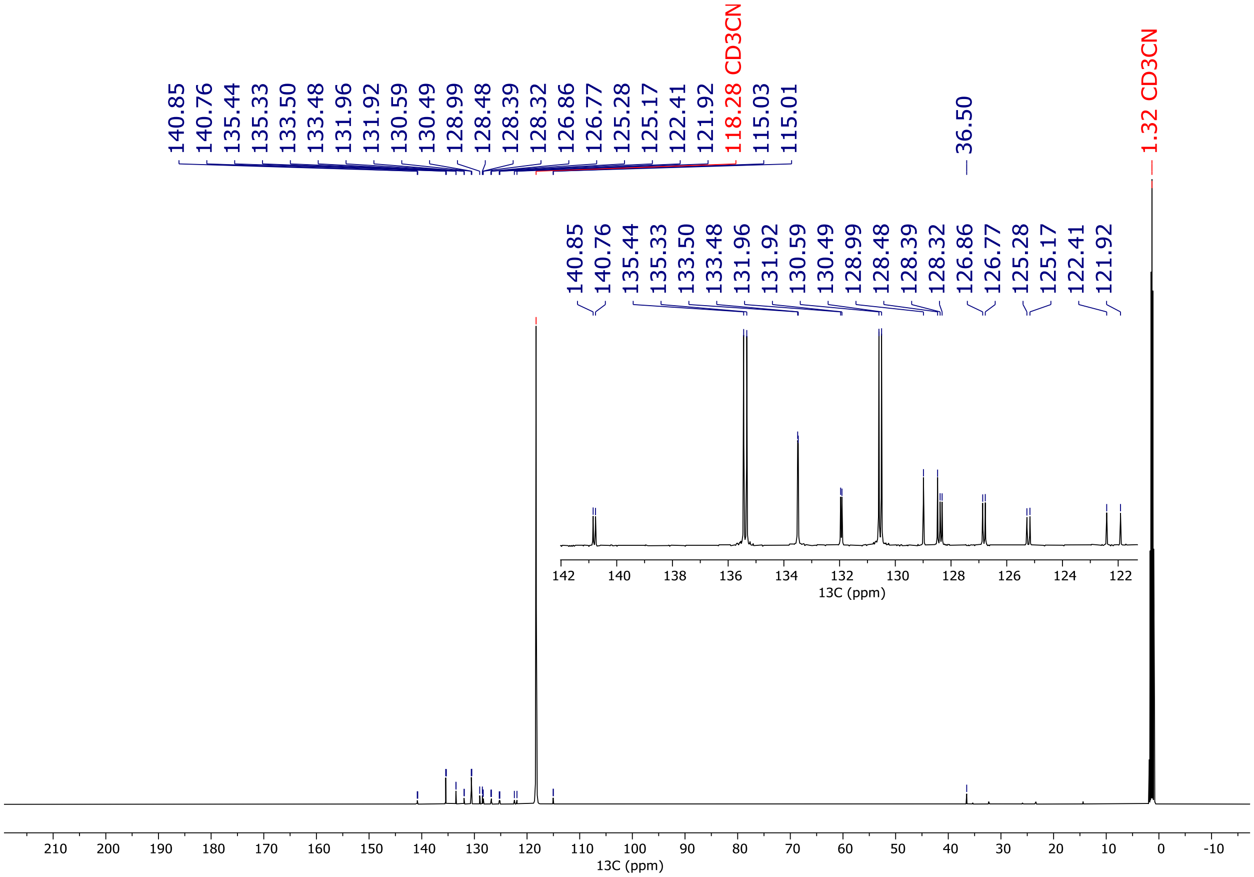

Figure S6. $^{13}C\{^1H\}$ **NMR** (125.77 MHz, CD_3CN) spectrum of **3**.

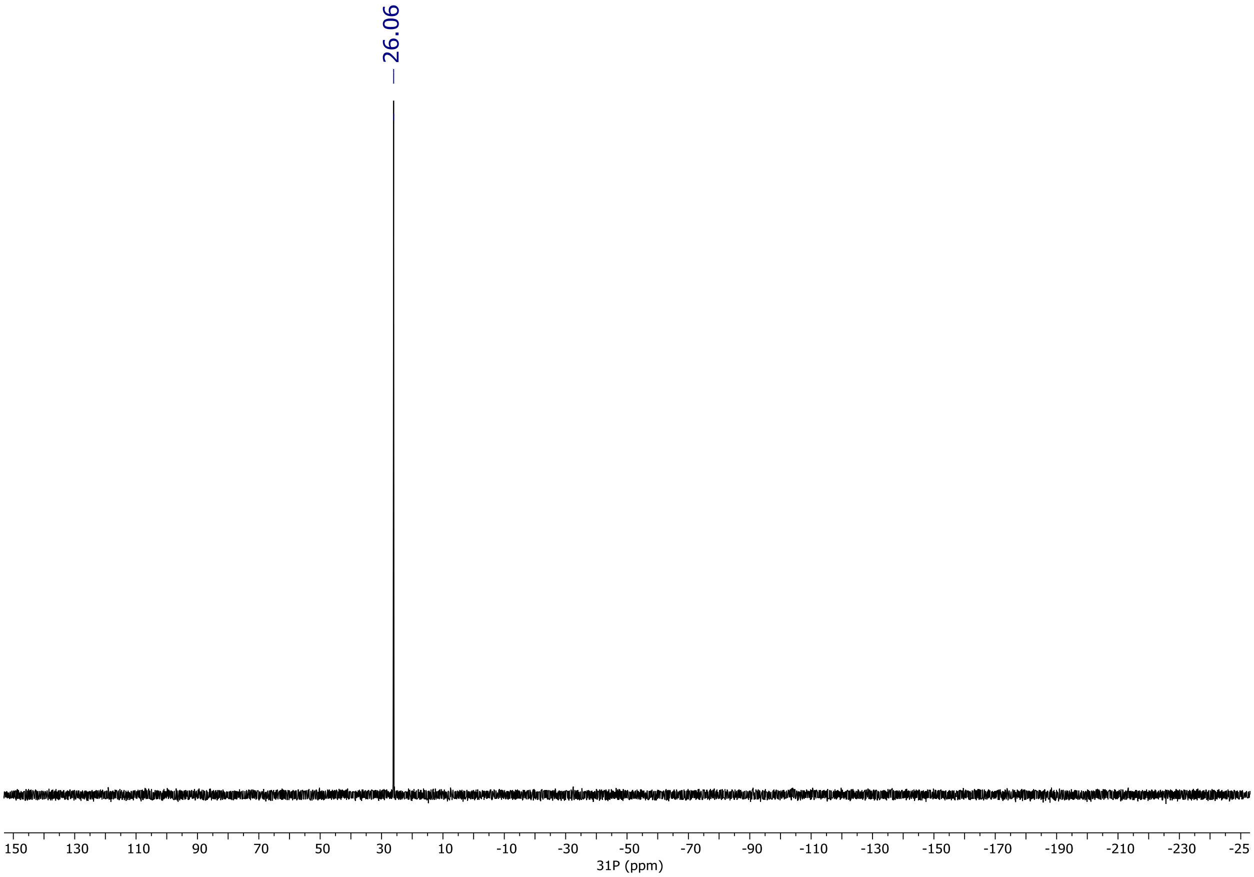

Figure S7. ^{31}**P{**^{1}**H} NMR** (162.00 MHz, CD_3CN) spectrum of **3**.

Synthesis of [4][OTf]

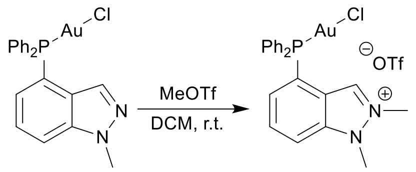

In a glove box, **3** (0.5120 g, 0.9330 mmol) was dissolved in dry DCM (2 mL) and methyl trifluoromethanesulfonate (0.1686 g, 1.027 mmol) was added dropwise with continuous stirring. The resulting mixture was stirred overnight at room temperature. The resulting solution was combined with hexanes (5 mL), resulting in the precipitation of [**4**][OTf] as a pale-yellow powder (0.5223 g, 0.7327 mmol, 79% yield). The powder was collected by filtration and further washed with Et_2O and hexanes. Colorless single crystals of [**4**][OTf] were obtained by slow diffusion of Et_2O into a concentrated solution of CH_3CN. **^{1}H NMR** (500.13 MHz, CD_3CN, 305.0 K) δ (ppm) 8.70 (s, 1H, indazole-N=C*H*), 7.97 (d, *J* = 8.9 Hz, 1H, indazole-*H*), 7.86 – 7.83 (m, 1H, indazole-*H*), 7.71 – 7.65 (m, 6H, Ph-*H*), 7.62 – 7.58 (m, 4H, Ph-*H*), 7.18 (dd, *J* = 13.7, 7.0 Hz, 1H, indazole-*H*), 4.19 (s, 6H, N-CH_3). **^{13}C{^{1}H} NMR** (125.77 MHz, CD_3CN, 305.0 K) δ (ppm) 141.70 (d, *J* = 10.0 Hz, *N-ipso*-indazole-*C*), 135.78 (d, *J* = 14.6 Hz, *o*-Ph-*C*), 134.16 (d, *J* = 2.6 Hz, *p*-Ph-*C*), 133.80 (d, *J* = 10.4 Hz, indazole-*C*), 132.95 (d, *J* = 5.8 Hz, indazole-*C*), 132.15 (d, *J* = 6.5 Hz, indazole-N=*C*H), 130.87 (d, *J* = 12.3 Hz, *m*-Ph-*C*), 127.03 (d, *J* = 64.9 Hz, *ipso*-Ph-*C*), 125.57 (d, *J* = 59.6 Hz, *ipso*-indazole-*C*), 120.46 (d, *J* = 14.1 Hz, indazole-*C*), 116.02 (d, *J* = 2.6 Hz indazole-*C*), 39.24 (s, N-CH_3), 34.72 (s, N-CH_3). **^{31}P{^{1}H} NMR** (162.00 MHz, CD_3CN, 298.0 K) δ (ppm) 24.97 (s). **^{19}F NMR** (376.42 MHz, CD_3CN, 298.0 K) δ (ppm) -79.32 (s) **Elemental Analysis:** $C_{22}H_{20}AuClF_3N_2O_3PS$: Calculated: C, 37.07; H, 2.83; N, 3.93; Found: C, 36.80; H, 2.75; N, 3.76.

NMR Spectra of [4][OTf]

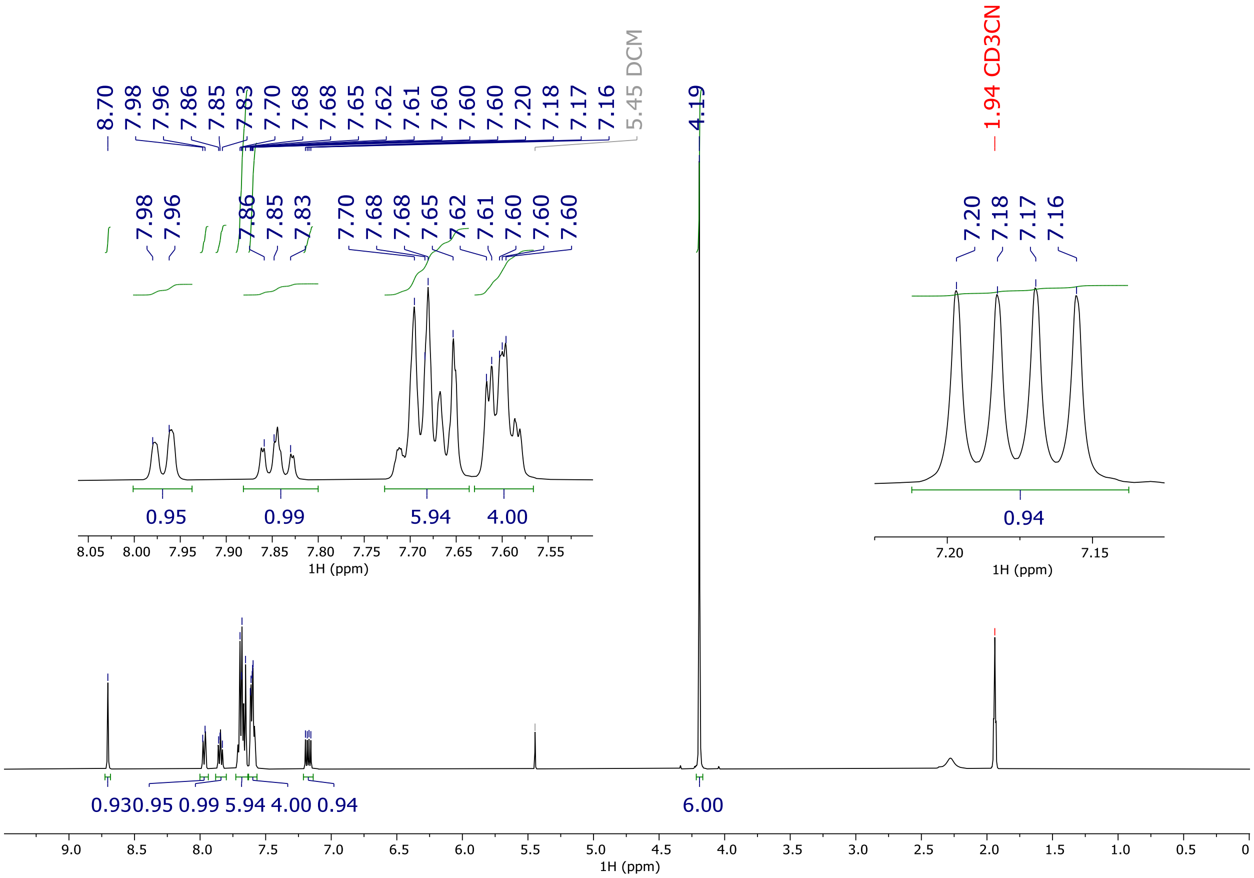

Figure S8. **^{1}H NMR** (500.13 MHz, CD_3CN) spectrum of [**4**][OTf].

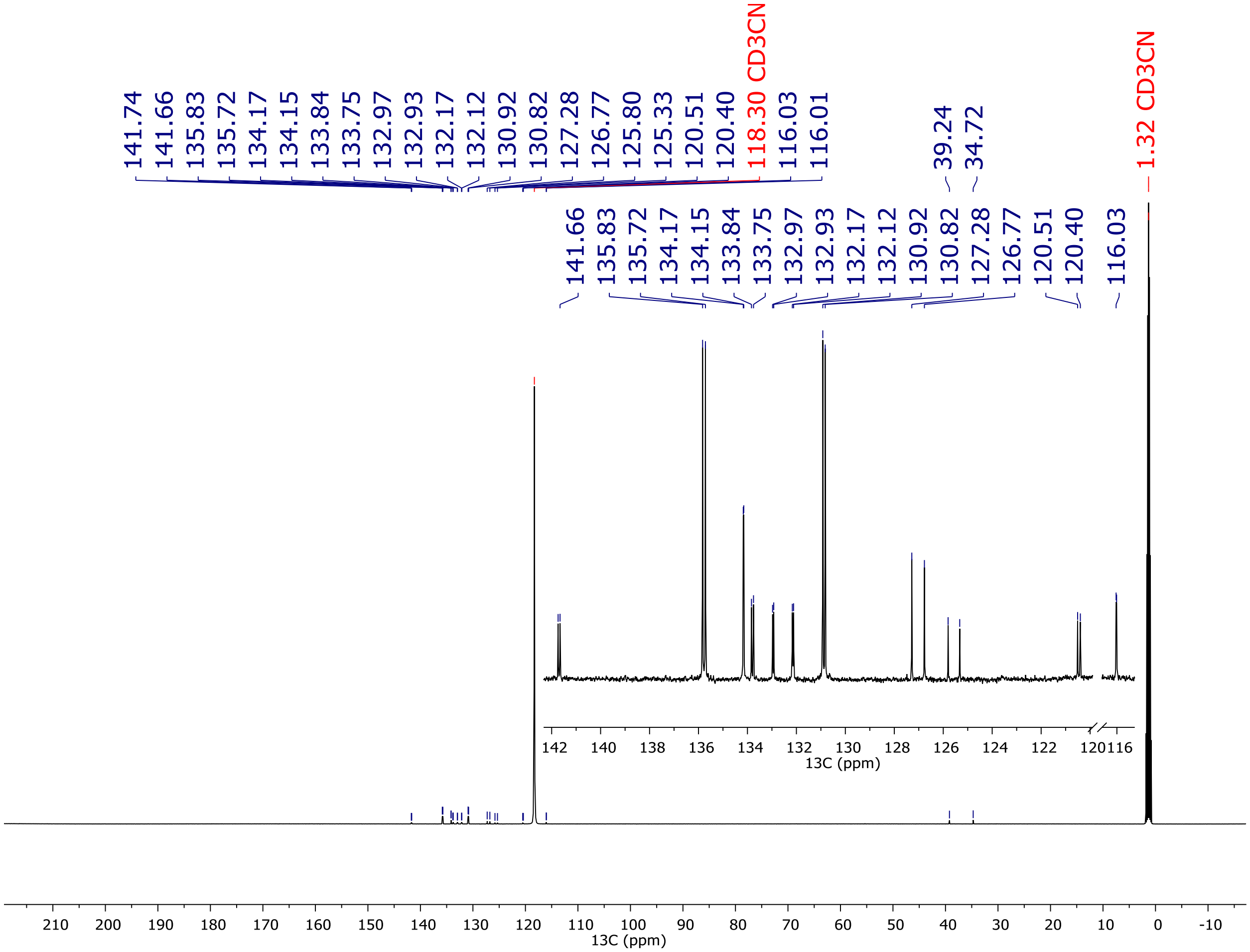

Figure S9. ^{13}**C{**^{1}**H} NMR** (125.77 MHz, CD_3CN) spectrum of [**4**][OTf].

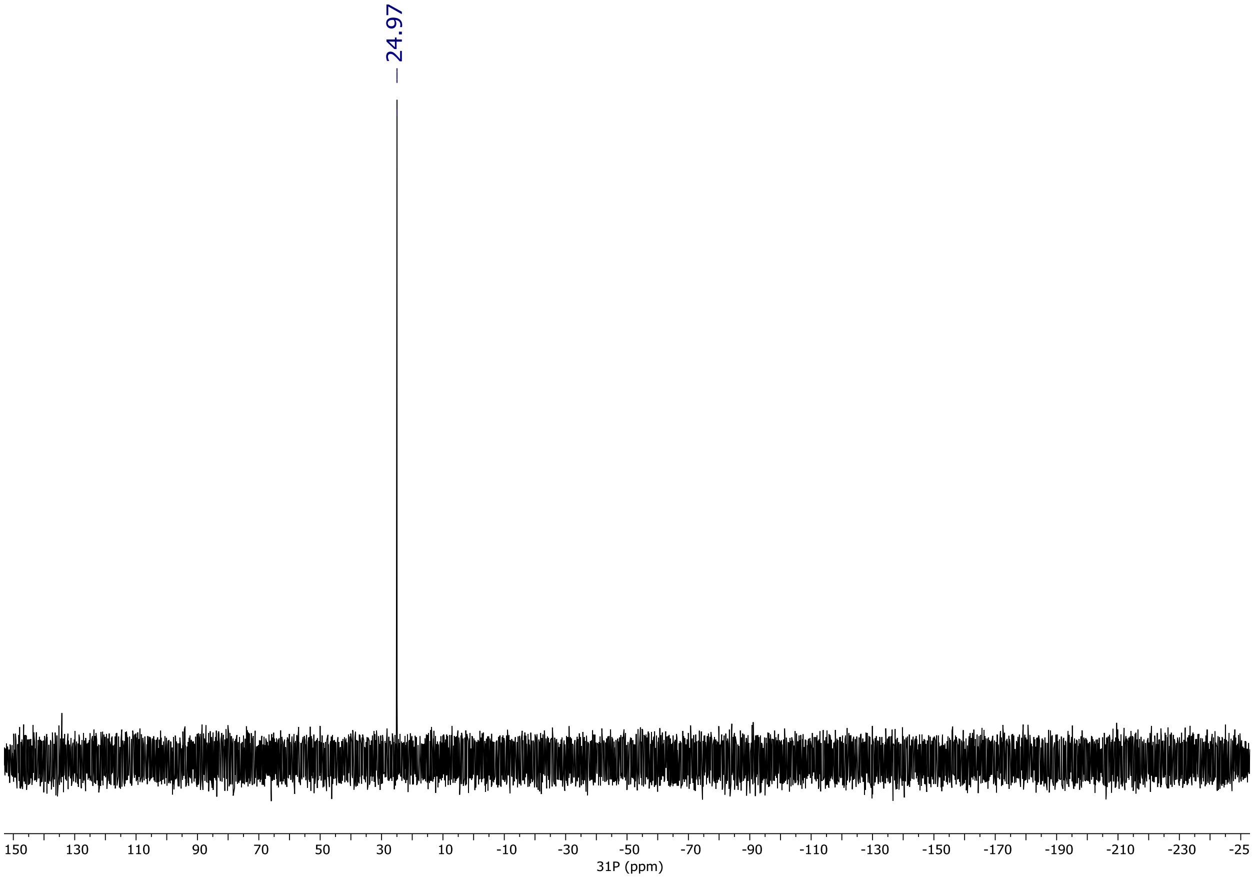

Figure S10. **$^{31}P\{^{1}H\}$ NMR** (162.00 MHz, CD_3CN) spectrum of [**4**][OTf].

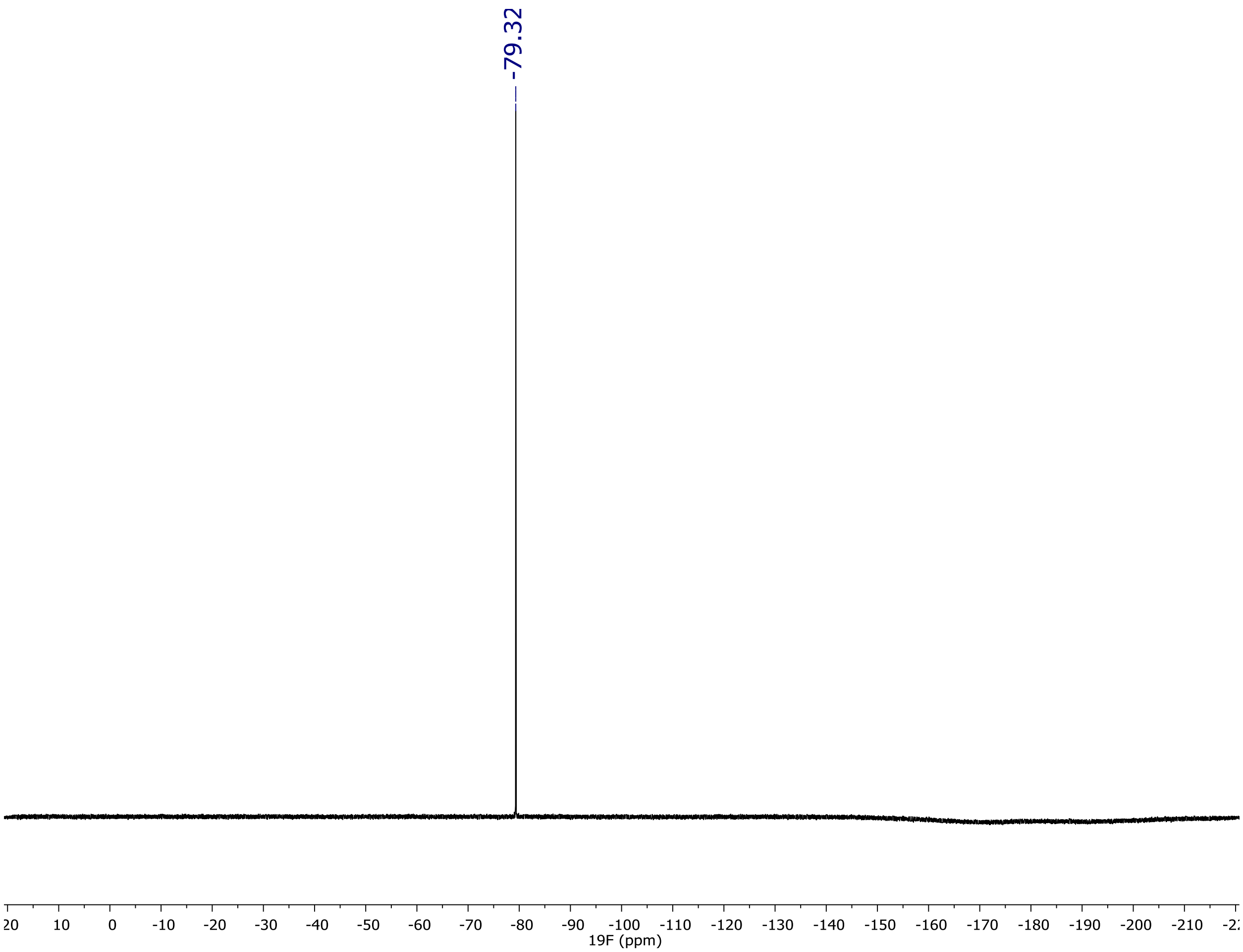

Figure S11. **^{19}F NMR** (376.42 MHz, CD_3CN) spectrum of [**4**][OTf].

Catalytic Studies

Procedure for Propargyl Amide Cyclization Studies

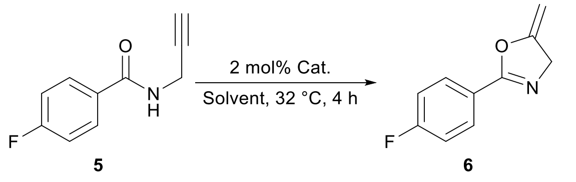

For the catalytic studies of propargyl amide cyclization, *N*-propargyl-4-fluorobenzamide (42.5 mg, 0.240 mmol) was added to a 20 mL vial with 2 mol% of crystallized catalyst. A solution of $CDCl_3$ (0.660 mL) and HFIP (60.0 µL) were then added to the 20 mL vial to dissolve the reaction mixture. An aliquot of 0.600 mL of this mixture was then added to an NMR tube, and the reaction progress was monitored *in situ* via ^{1}H NMR. Spectra were recorded every 20 minutes. Final conversion of the reaction was measured based on the integrated ^{1}H NMR spectra. The same procedure was followed for the trials without HFIP, but the volume of $CDCl_3$ was adjusted to 0.720 mL to maintain the same reaction concentration. **^{1}H NMR** (500.13 MHz, $CDCl_3$, 305.0 K) δ (ppm) 7.97 (dd, *J* = 8.8, 5.3 Hz, 2H, Ar-H), 7.15 (t, *J* = 17.3 Hz, 2H, Ar-H), 4.91 (q, J = 3.3 Hz, 1H, =CH_2), 4.60 (t, *J* = 6.0 Hz, 2H, CH_2), 4.47 (q, *J* = 2.9 Hz, 1H, =CH_2). The results of these catalyses are presented in **Table 1** in the main text.

Stacked Spectra for Propargyl Amide Cyclization Studies

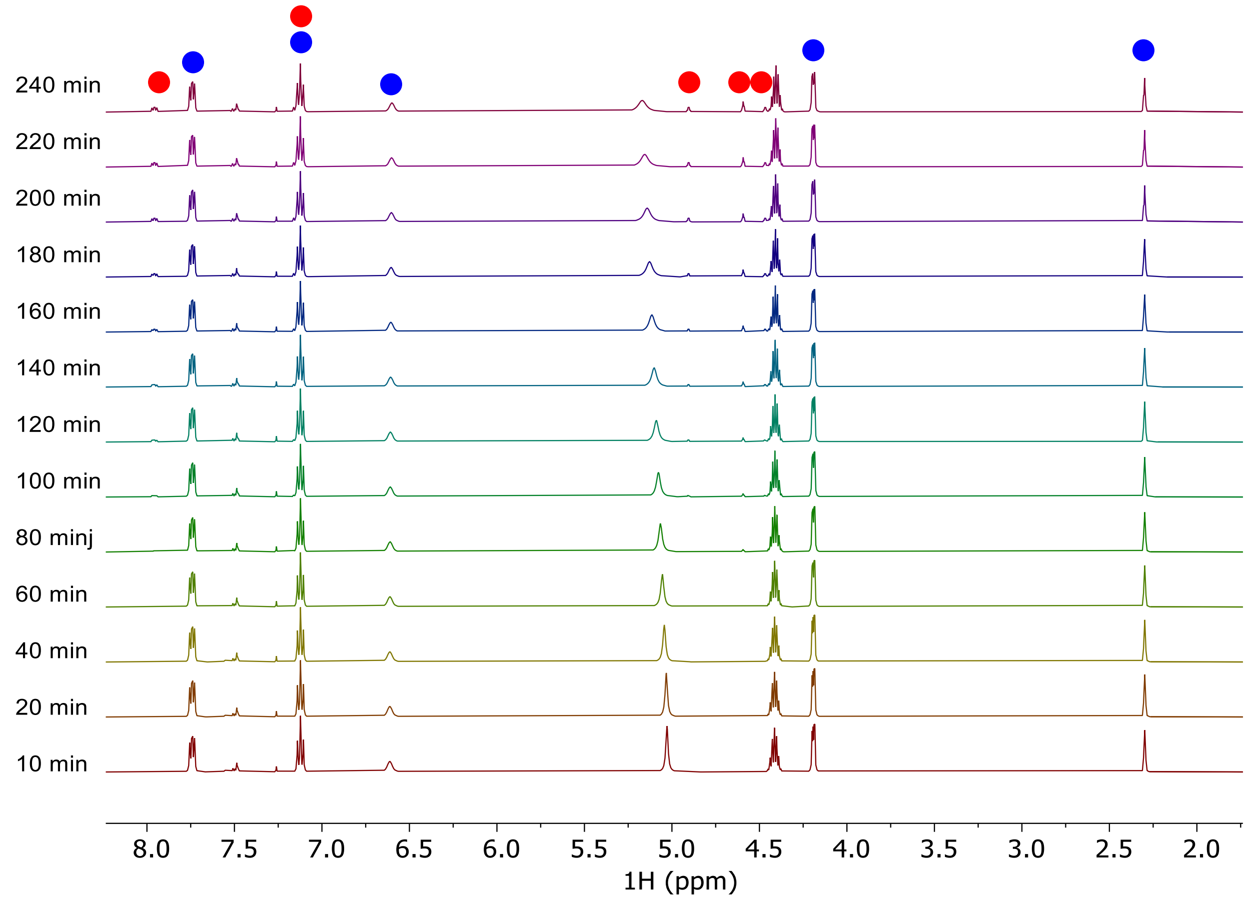

Figure S12. Stacked ^{1}H NMR spectra (500.13 MHz, $CDCl_3$, 305.0 K) collected in situ during the reaction corresponding to Entry 1 in **Table 1** of the main text. Starting material (**5**): blue circle; Product (**6**): red circle

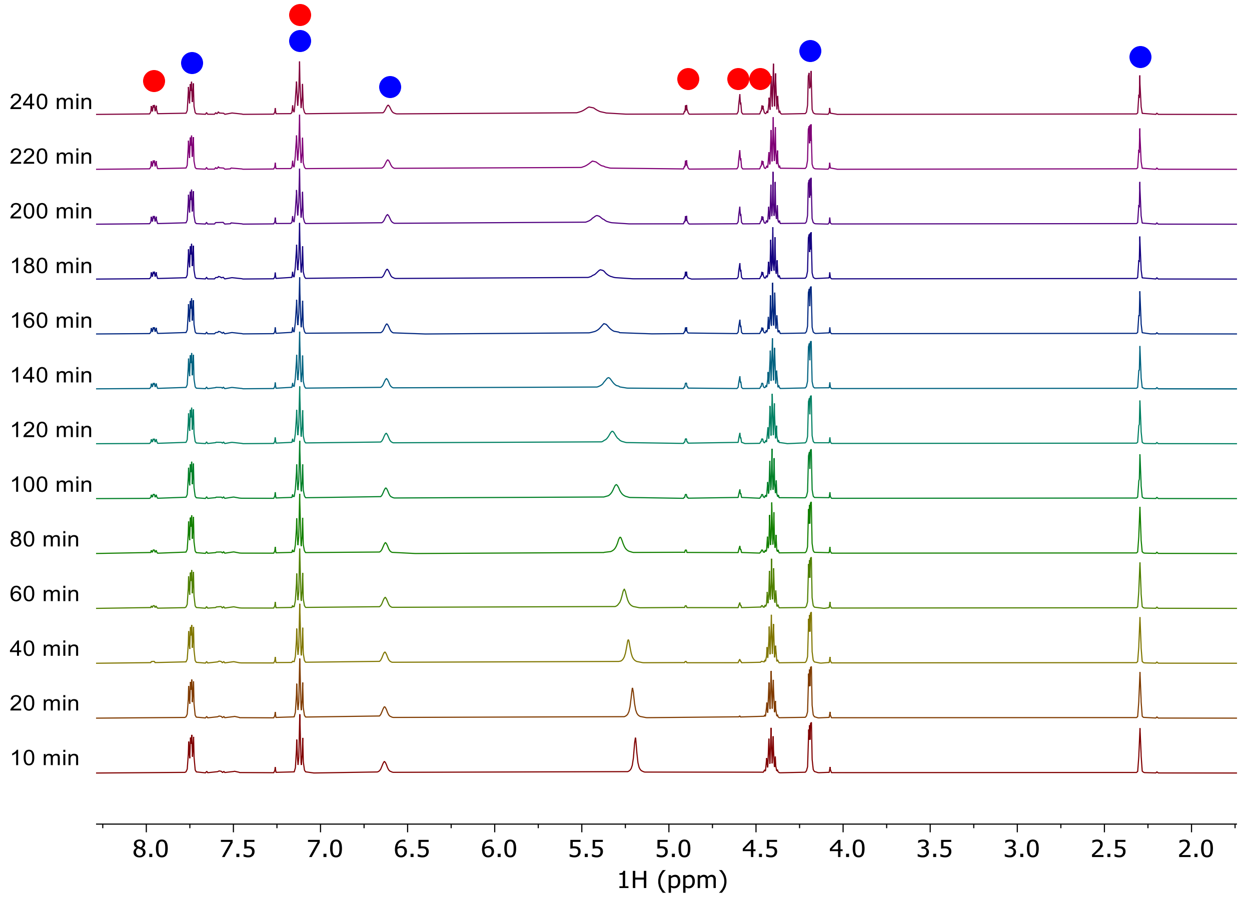

Figure S13. Stacked ^{1}H NMR spectra (500.13 MHz, $CDCl_3$, 305.0 K) collected in situ during the reaction corresponding to Entry 2 in **Table 1** of the main text. Starting material (**5**): blue circle; Product (**6**): red circle

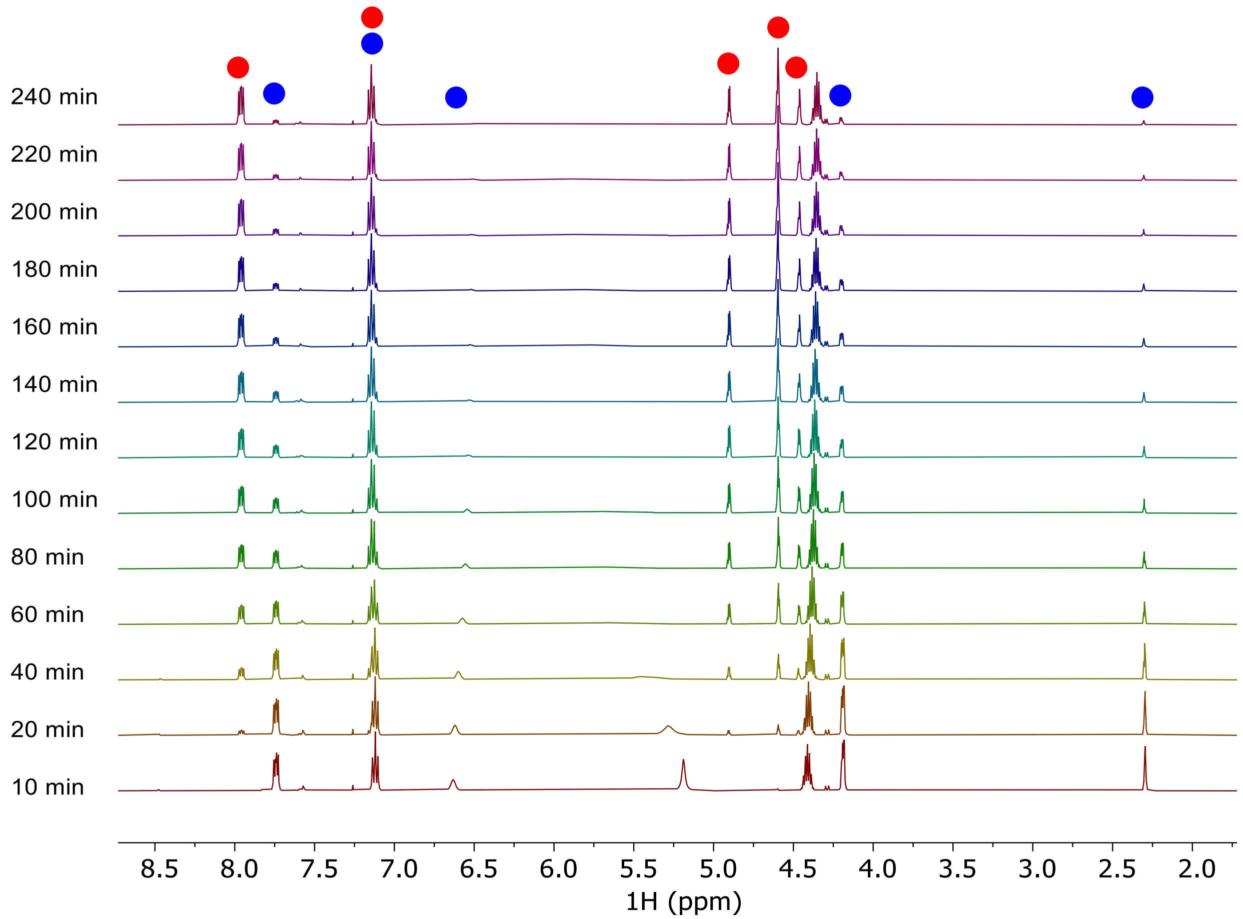

Figure S14. Stacked ^{1}H NMR spectra (500.13 MHz, $CDCl_3$, 305.0 K) collected in situ during the reaction corresponding to Entry 3 in **Table 1** of the main text. Starting material (**5**): blue circle; Product (**6**): red circle

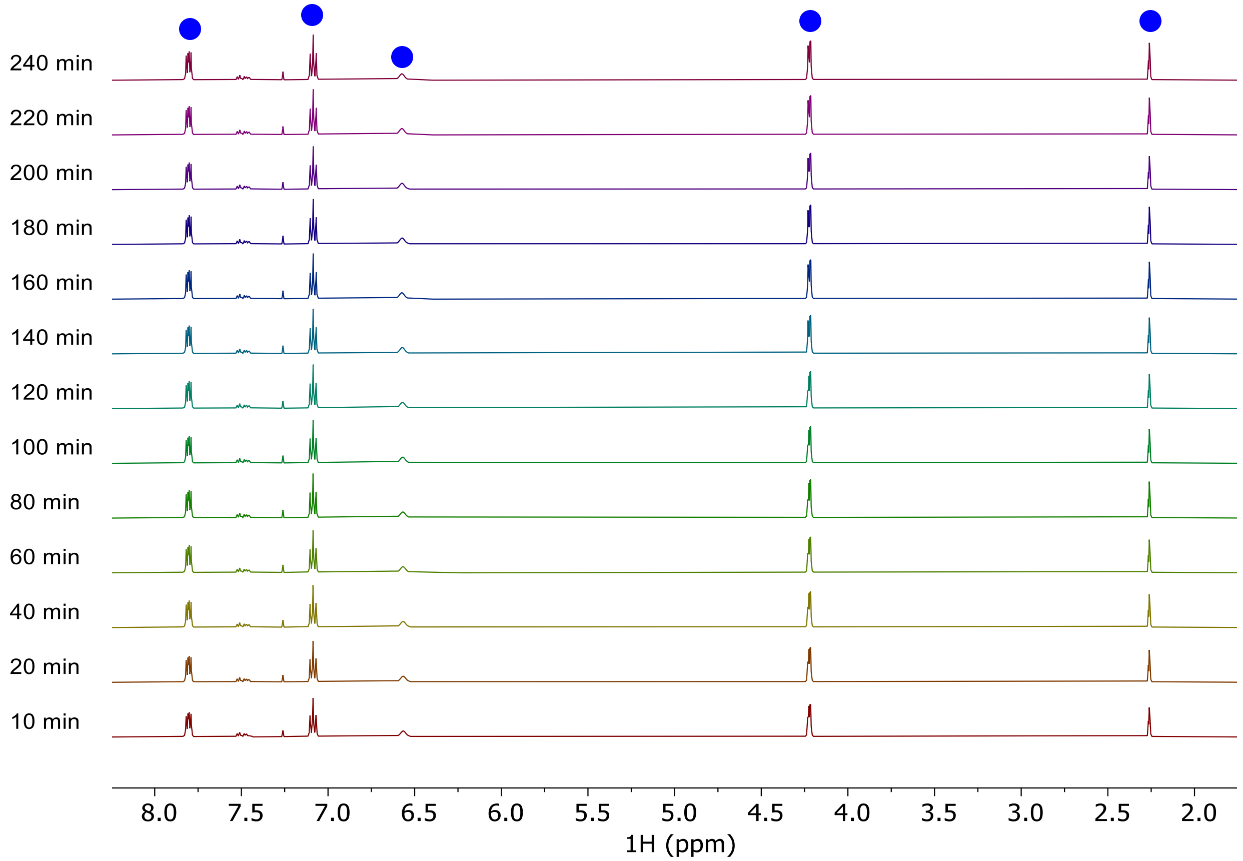

Figure S15. Stacked ^{1}H NMR spectra (500.13 MHz, $CDCl_3$, 305.0 K) collected in situ during the reaction corresponding to Entry 4 in **Table 1** of the main text. Starting material (**5**): blue circle

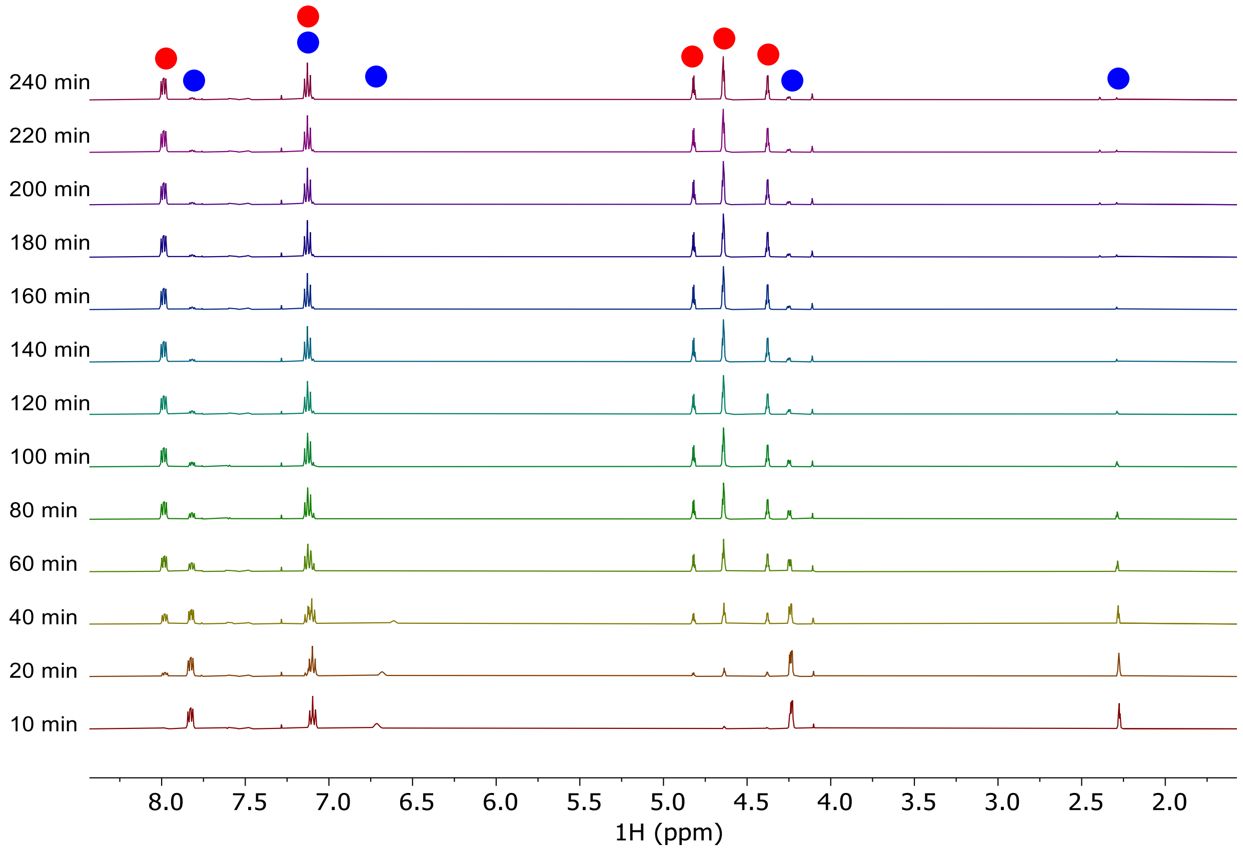

Figure S16. Stacked ^{1}H NMR spectra (500.13 MHz, $CDCl_3$, 305.0 K) collected in situ during the reaction corresponding to Entry 5 in **Table 1** of the main text. Starting material (**5**): blue circle; Product (**6**): red circle

Procedure for Enyne Cyclization Studies

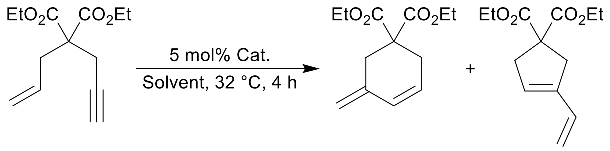

7 **8a** **8b**

For the catalytic studies of enyne cyclization, 2-allyl-2-(2-propynyl)malonate (71.5 mg, 0.300 mmol) was added to a 20 mL vial with 5 mol% of crystallized catalyst. A solution of $CDCl_3$ (0.600 mL) and HFIP (0.300 mL) were then added to the 20 mL vial to dissolve the reaction mixture. An aliquot of 0.600 mL of this mixture was then added to an NMR tube, and the reaction progress was monitored *in situ* via ^{1}H NMR. Spectra were recorded every 20 minutes. Final conversion of the reaction was measured based on the integrated ^{1}H NMR spectra. The same procedure was followed for the trials without HFIP, but the volume of $CDCl_3$ was adjusted to 0.900 mL to maintain the same reaction concentration. **8a: ^{1}H NMR** (500.13 MHz, $CDCl_3$, 305.0 K) δ (ppm) 6.15 (d, J = 9.9 Hz, 1H), 5.77 – 5.73 (m, 1H), 4.94 (d, J = 14.0 Hz, 2H), 4.20 (m, 4H), 2.84 (t, J = 1.6 Hz, 2H), 2.64 (dd, J = 4.4, 2.1 Hz, 2H), 1.26 (m, 6H). **8b: ^{1}H NMR** (500.13 MHz, $CDCl_3$, 305.0 K) δ (ppm) 6.48 (dd, *J* = 17.5, 10.8 Hz, 1H), 5.60 (t, J = 2.4 Hz, 1H), 5.15 – 5.12 (m, 2H), 4.22 (m, 4H), 3.11 – 3.08 (d, J = 13.4 Hz, 4H), 1.27 (m, 6H).

Stacked Spectra for Enyne Cyclization Studies

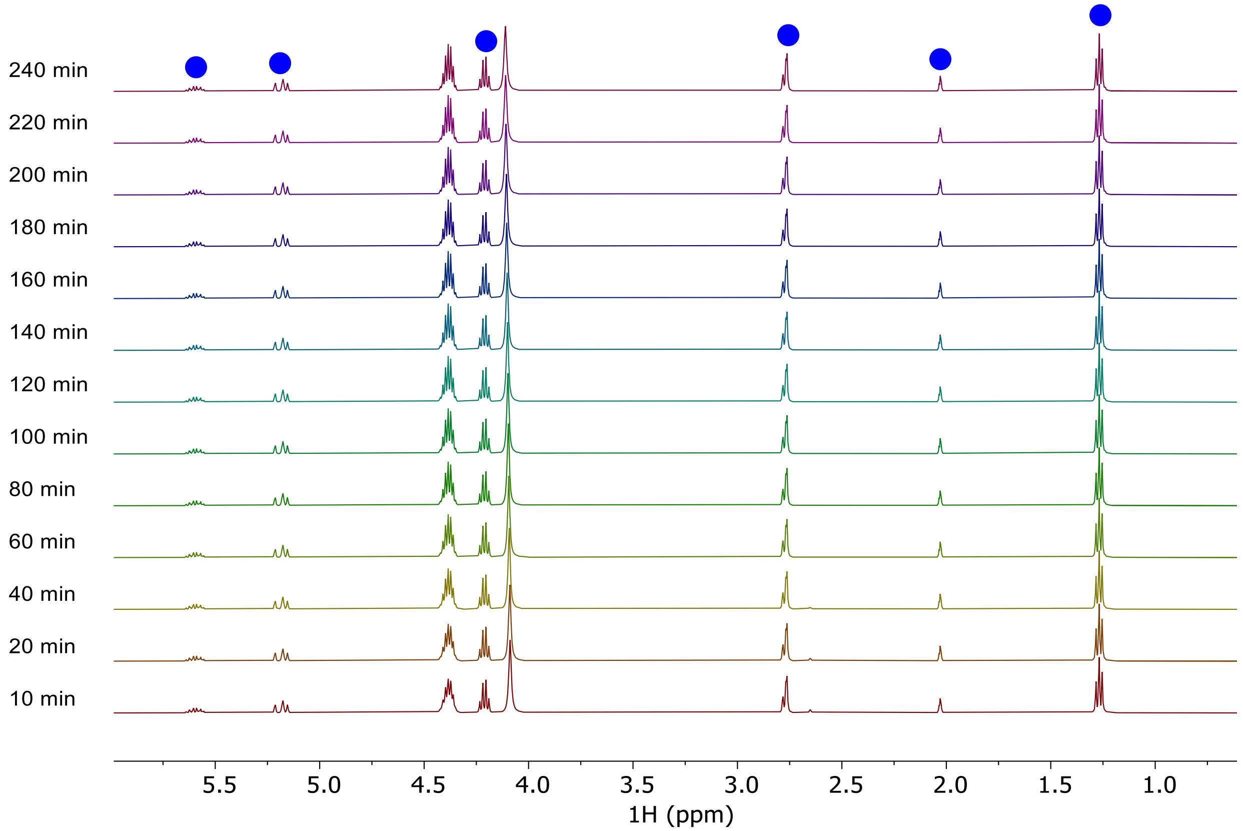

Figure S17. Stacked ^{1}H NMR spectra (500.13 MHz, $CDCl_3$, 305.0 K) collected in situ during the reaction corresponding to Entry 1 in **Table 2** of the main text. Starting material (**7**): blue circle

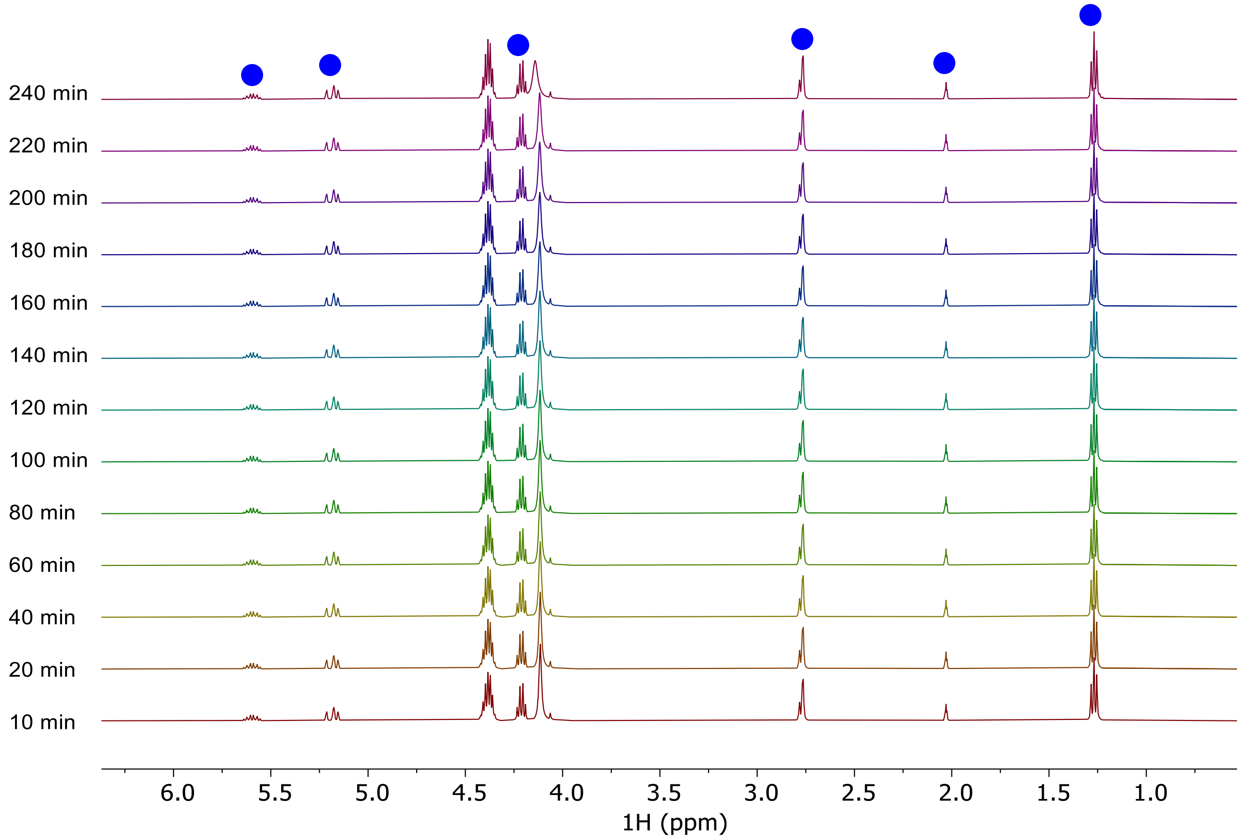

Figure S18. Stacked ^{1}H NMR spectra (500.13 MHz, $CDCl_3$, 305.0 K) collected in situ during the reaction corresponding to Entry 2 in **Table 2** of the main text. Starting material (**7**): blue circle

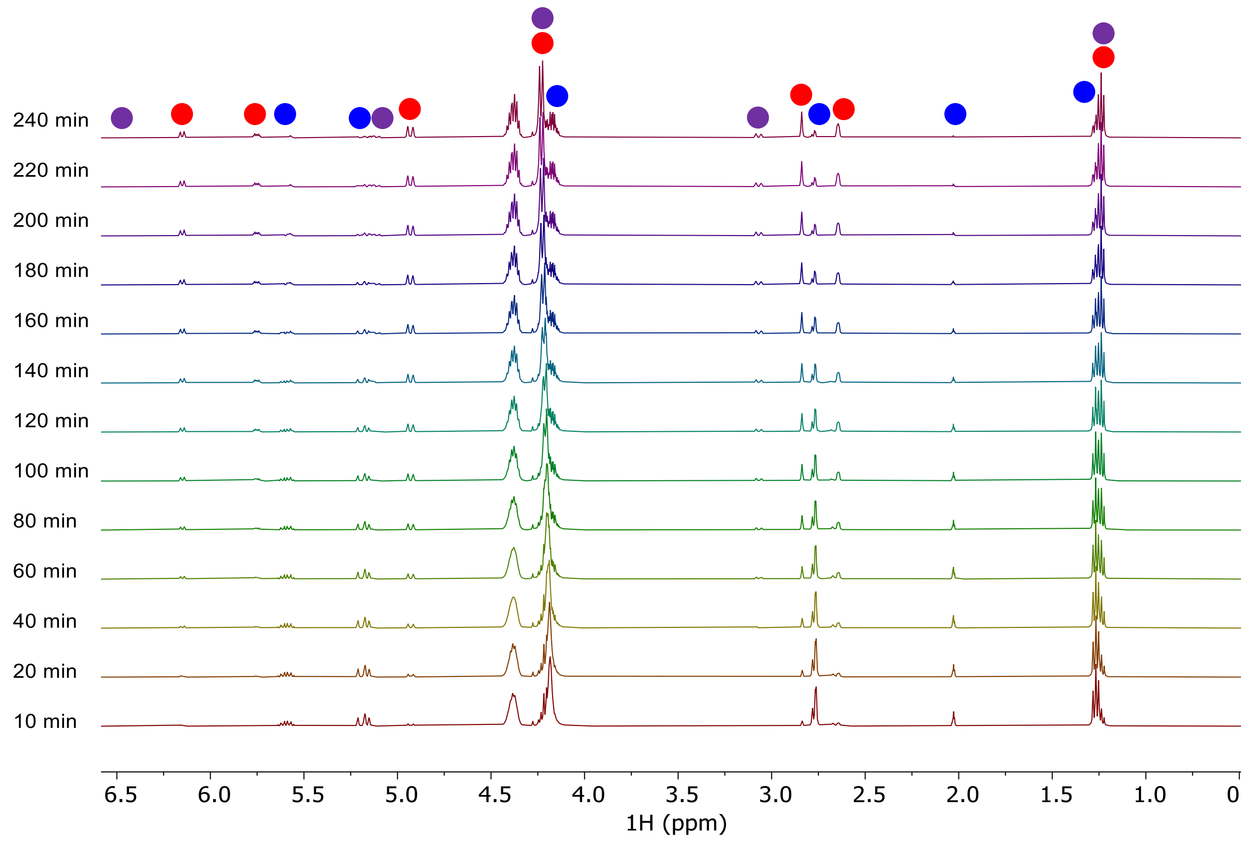

Figure S19. Stacked ^{1}H NMR spectra (500.13 MHz, $CDCl_3$, 305.0 K) collected in situ during the reaction corresponding to Entry 3 in **Table 2** of the main text. Starting material (**7**): blue circle; Product (**8a**): red circle; Product (**8b**): purple circle

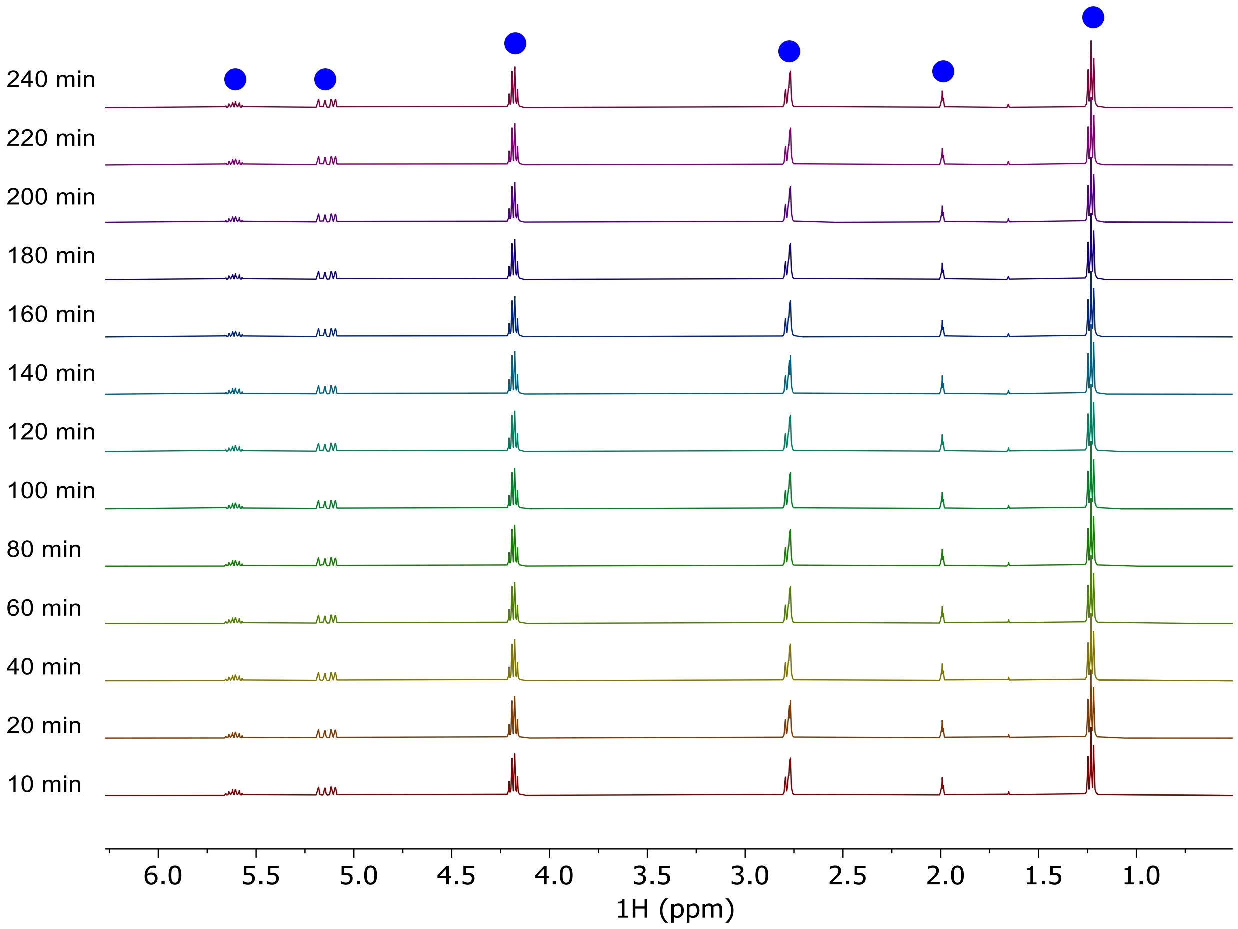

Figure S20. Stacked ^{1}H NMR spectra (500.13 MHz, $CDCl_3$, 305.0 K) collected in situ during the reaction corresponding to Entry 4 in **Table 2** of the main text. Starting material (**7**): blue circle

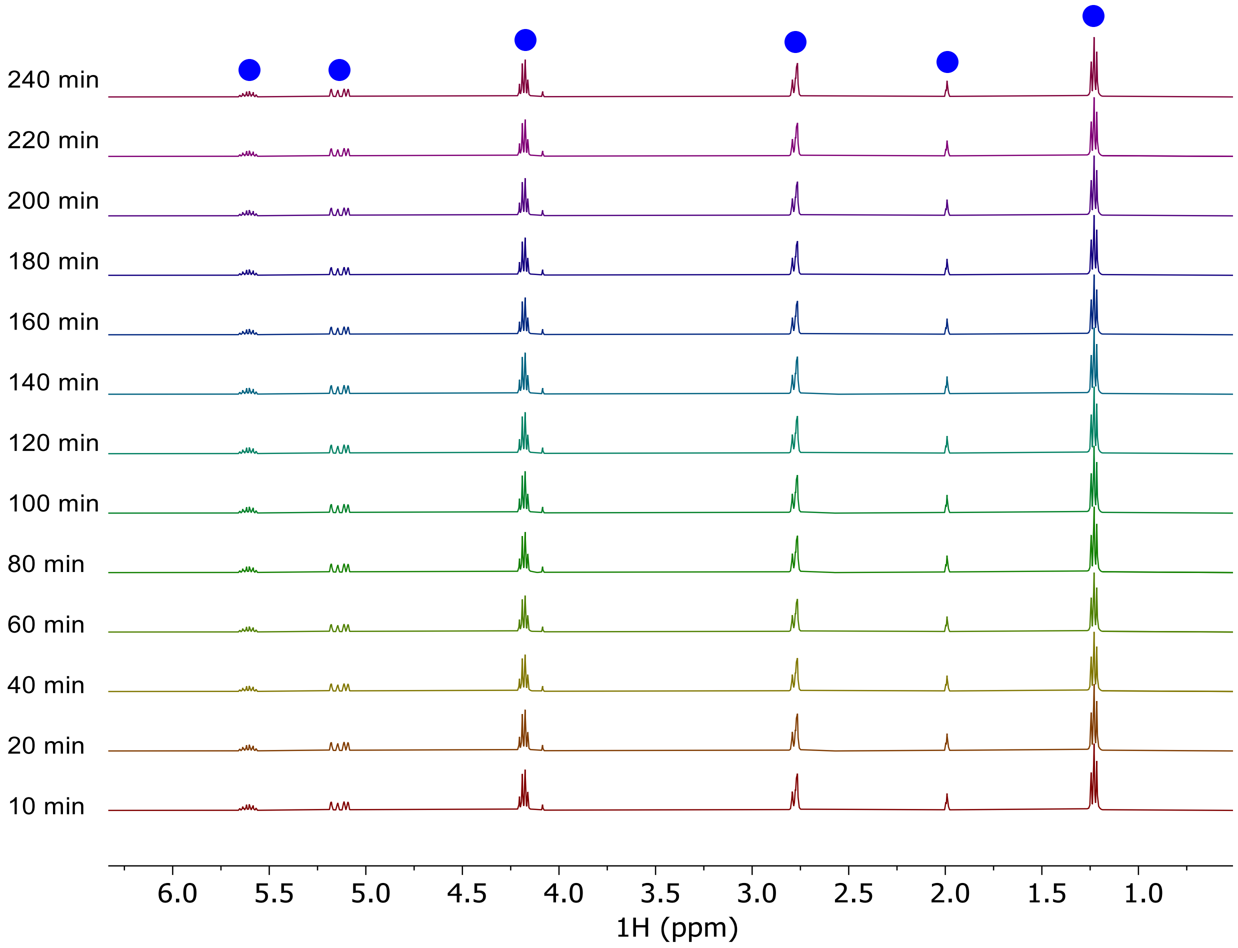

Figure S21. Stacked ^{1}H NMR spectra (500.13 MHz, $CDCl_3$, 305.0 K) collected in situ during the reaction corresponding to Entry 5 in **Table 2** of the main text. Starting material (**7**): blue circle

References

1. Carreras, J.; Pereira, A.; Zanini, M.; Echavarren, A. M., Variations on the Theme of JohnPhos Gold(I) Catalysts: Arsine and Carbene Complexes with Similar Architectures. *Organometallics* **2018,** *37*, 3588-3597.
2. Uson, R.; Laguna, A.; Laguna, M., Tetrahydrothiophene gold(I) or gold(III) complexes. *Inorganic Syntheses* **1989,** *26*, 85-91.
3. *APEX4*; Bruker AXS Inc.: Madison, Wisconsin, USA. **2021**.
4. Sheldrick, G. M., *SADABS, Version 2007/4, Bruker Analytical X-ray Systems, Inc., Madison, Wisconsin, USA*. 2007.
5. Sheldrick, G. M., SHELXT - integrated space-group and crystal-structure determination. *Acta Crystallographica Section A* **2015,** *71*, 3-8.
6. Dolomanov, O. V.; Bourhis, L. J.; Gildea, R. J.; Howard, J. A. K.; Puschmann, H., OLEX2: a complete structure solution, refinement and analysis program. *Journal of Applied Crystallography* **2009,** *42*, 339-341.
